# Mitochondrion-targeted supramolecular “nano-boat” simultaneously inhibiting dual energy metabolism for tumor selective and synergistic chemo-radiotherapy

**DOI:** 10.7150/thno.67543

**Published:** 2022-01-01

**Authors:** Jie Gao, Zhilong Wang, Qingxiang Guo, Huan Tang, Zhongyan Wang, Cuihong Yang, Huirong Fan, Wenxue Zhang, Chunhua Ren, Jianfeng Liu

**Affiliations:** 1 Key Laboratory of Radiopharmacokinetics for Innovative Drugs, Institute of Radiation Medicine, Chinese Academy of Medical Sciences & Peking Union Medical College, Tianjin 300192, P. R. China.; 2 Radiation Oncology Department, Tianjin Medical University General Hospital, Tianjin 300052, P. R. China.

**Keywords:** cancer metabolism, peptide self-assembly, mitochondrion-targeted, selective killing, radio-sensitization

## Abstract

**Rationale:** Tumor energy metabolism has been a well-appreciated target of cancer therapy; however, the metabolism change of cancer cells between oxidative phosphorylation and glycolysis poses a challenge to the above. In this study, we constructed an innovative mitochondrion-targeted supramolecular “nano-boat” based on peptide self-assembly for tumor combined chemo-radiotherapy by simultaneously inhibiting the dual energy metabolism.

**Methods:** A lipophilic self-assembled peptide and a positively charged cyclen were integrated to fabricate a brand new mitochondrion-targeted nano-platform for the first time. The indices of mitochondrial dysfunction including mitochondrial membrane potential, apoptosis proteins expression and ultrastructure change were evaluated using a JC-1 probe, western blotting and biological transmission electron microscopy, respectively. Energy metabolism assays were conducted on a Seahorse XF24 system by detecting the oxygen consumption rate and the glycolytic proton efflux rate. The radio-sensitization effect was investigated by colony formation, the comet assay, and γ-H2AX staining.

**Results:** The supramolecular “nano-boat” could selectively kill cancer cells by much higher enrichment and reactive oxygen species generation than those in normal cells. In the cancer cells treated with the supramolecular “nano-boat”, the dysfunctional morphological changes of the mitochondrial ultrastructure including swelling and pyknosis were evidently observed, and the endogenous mitochondrial apoptosis pathway was effectively triggered by abundant of cytochrome C leaking out. Concurrently, the dual metabolic pathways of glycolysis and oxidative phosphorylation were severely inhibited. More importantly, the supramolecular “nano-boat” displayed an excellent radio-sensitization effect with a sensitization enhancement ratio value as high as 2.58, and hence, *in vivo* efficiently combining radiotherapy yielded an enhanced chemo-radiotherapy effect.

**Conclusion:** Our study demonstrated that the rationally designed peptide-based “nano-boat” could efficiently induce cancer cell apoptosis by the energy metabolism inhibition involving multiple pathways, which may provide the motivation for designing novel and universal mitochondria-targeted drug delivery systems for cancer therapy.

## Introduction

Tumor metabolism has been extensively exploited as a therapeutic target 1, 2. Distinct from normal cells, most cancer cells adopt aerobic glycolysis to generate adenosine triphosphate (ATP) for meeting the high demand of energy and biomass production, which is also known as the Warburg effect 3. Such a metabolic reprogramming has become a well-appreciated hallmark of cancer therapy, owing to that glycolysis has been proved as the energy basis of oncogene growth, tumor development, and metastasis 4-6. Therefore, in numerous studies, many drugs that target the enzymes and transporters involved in glucose metabolism, such as hexokinase (HK), lactate dehydrogenase A (LDHA), glucose transporter 1 (GLUT1), and monocarboxylate transporter 1 (MCT1), had been utilized to inhibit glycolysis for cancer therapy 7-11. Concurrently, the emerging studies presently have shown that mitochondrial metabolism is also indispensable for cancer cell survival, growth and proliferation. Many cancer cells have the ability to convert their metabolic phenotype between oxidative phosphorylation (OXPHOS) and glycolysis for adapting to an external altered environment 12-14. Additionally, glycolysis and OXPHOS are also metabolically coupled and cooperated closely via the biosynthesis pathways to generate cellular components 15-17. Hence, mitochondrion-targeted drug delivery systems simultaneously inhibiting the dual energy metabolism hold great potential for anti-tumor therapy.

The notable difference in the mitochondrial membrane potential (MMP) of tumor cells and normal cells has provided an intrinsic basis for mitochondrion-targeted cancer therapy 18-21. The MMP depolarization can cause the release of cytochrome C and Smac proteins from the mitochondrial membrane, which can subsequently activate the caspase-related apoptosis pathway and finally kill cancer cells 22-26. In addition, the increase in the oxidative stress caused by mitochondrial dysfunction is also closely related to the radio-sensitivity of cancer cells 27-29. However, owing to the hydrophobic double membrane structure and the negative potential, it is challenging for most drugs to access in the mitochondrion. Although the triphenylphosphonium (TPP) and some penetrating peptides are commonly employed for constructing mitochondrion-targeted delivery systems 30-32, a simple and universal mitochondrion-targeted nano-platform is highly demanded. The cationic charge and lipophilicity have been commonly confirmed as the two critical factors 33-36, providing the key design basis for novel mitochondrion-targeted systems. It has been reported that macrocyclic amines (e.g., cyclen) are more easily protonated in the acidic condition in the tumor and further bind with negatively charged molecules 37, 38. In addition, the hydrophobic short peptides forming self-assembled nanomedicine have shown major promise in improving drug delivery for cancer chemotherapy 39-41, radiotherapy 42, photodynamic therapy 43 and immunotherapy 44 as well. Utilizing the advantages of the cationic charge of macrocyclic amines and the lipophilicity of hydrophobic self-assembled peptides, a conjugate resulting from their integration can be expected to serve as an ideal nano-platform for mitochondrion-targeted drug delivery.

**Scheme 1:**
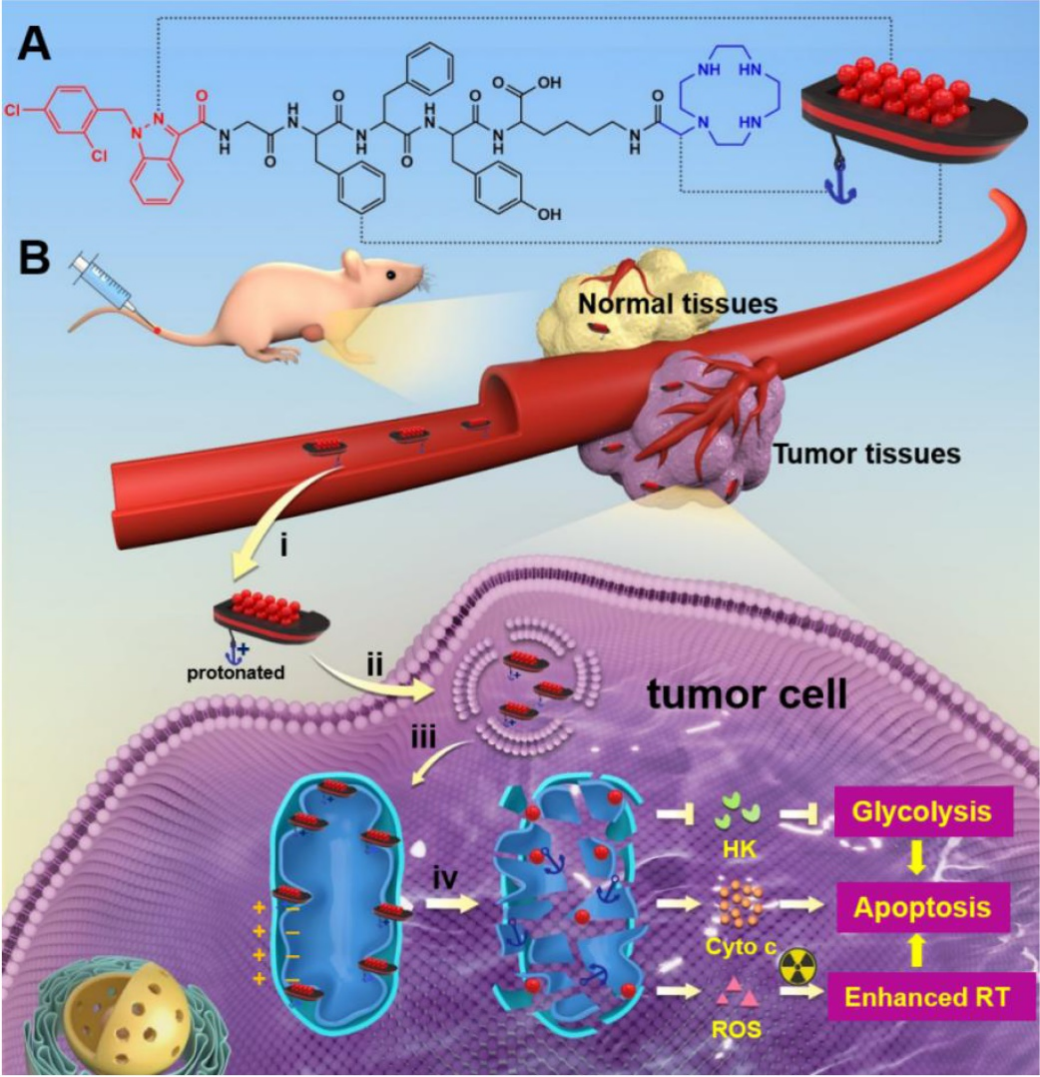
** (A)** Molecular chemical structure of LND-GFFYK-cyclen and schematic illustration of the drug-peptide conjugates self-assemble into LND-loaded supramolecular “nano-boat”. **(B)** Schematic illustration of intravenous injected supramolecular “nano-boat” for *in vivo* tumor selective and combined chemo-radiotherapy by targeting and damaging mitochondrion: i) enhanced permeability and retention (EPR) effect; ii) internalization; iii) release and target; iv) mitochondrial dysfunction. (HK: hexokinase; Cyto c: cytochrome C; RT: radiotherapy)

Lonidamine (LND) is a potential energy stripper of cancer cells, which can not only inhibit glycolysis by suppressing the activity of HK-II, but also can impede OXPHOS by destroying the mitochondrial structure 45-47. However, the clinical application of LND is limited by its low bio-availability and severe hepatotoxicity 48. In this study, we aimed to construct a brand new nano-platform targeting LND into the mitochondrion for selective and combined cancer chemo-radiotherapy. The designed LND-loaded nano-platform could be visualized as a supramolecular “nano-boat”, which was composed with three parts (Scheme 1). These were a short peptide chain (GFFYK) constituting the main body of the “nano-boat”, LND serving as an “aggressive cargo” as well as an indispensable part of the “nano-boat”, and cyclen being used as the anchor of the “nano-boat”. After tail vein injection into the tumor-bearing mice, the “nano-boats” entering the blood circulation showed much higher accumulation in tumor tissues than in normal tissues owing to the protonation in the acidic pH condition. On being internalized and released from the inclusion body, the protonated “nano-boats” sailed and anchored to the mitochondria for selectively attacking the cancer cells, which eventually simultaneously inhibited the dual energy metabolism and combined with the radiotherapy inducing endogenous apoptosis pathway of tumor cells.

## Results and Discussion

### Preparation and characterization of supramolecular “nano-boat”

**Figure 1:**
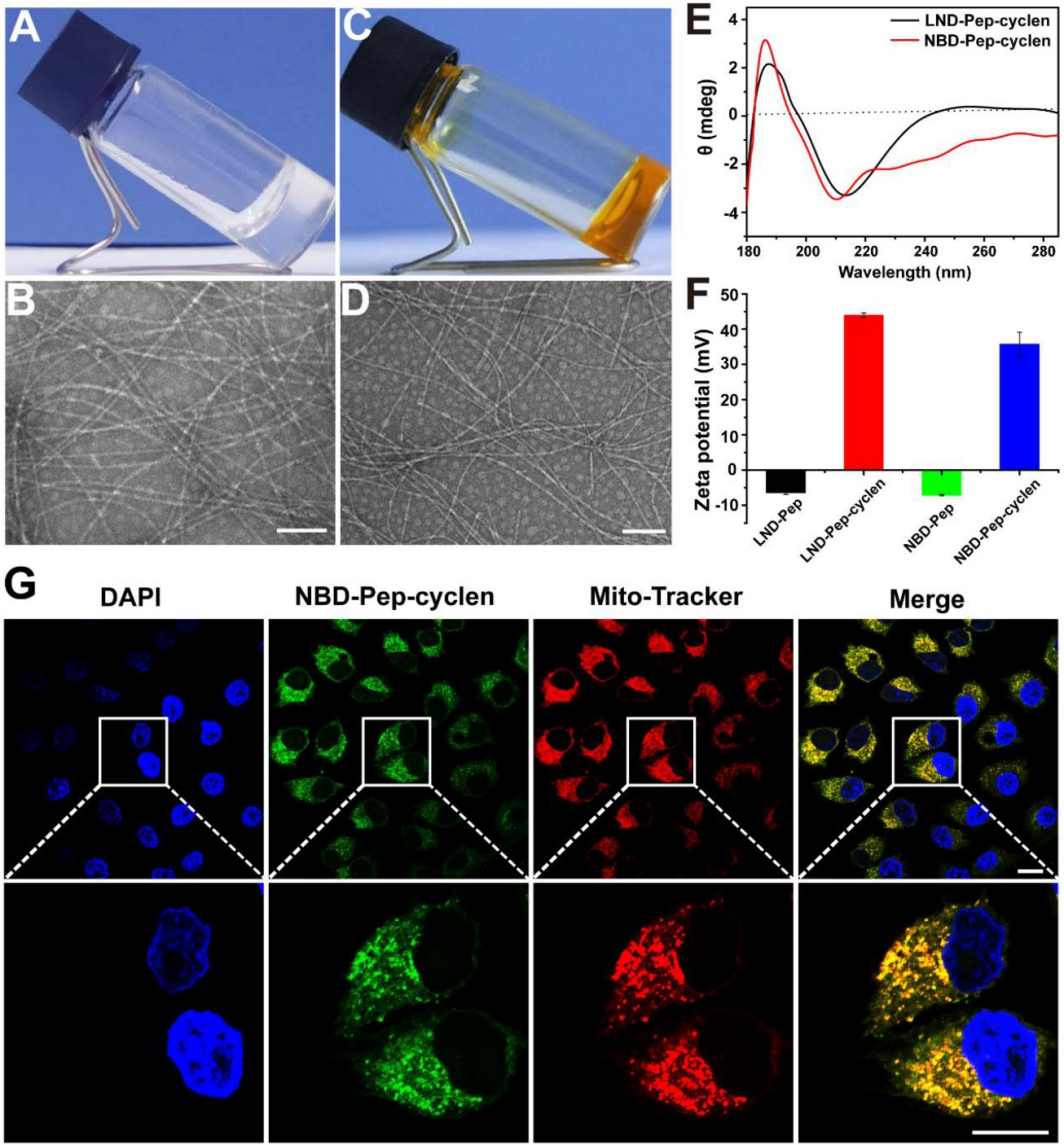
**Characterization of the supramolecular “nano-boat”.** Optical images of hydrogels formed by LND-Pep-cyclen **(A)** and NBD-Pep-cyclen **(C)** at the concentration of 4 mg/mL. Representative TEM images of the hydrogels formed by **(B)** LND-Pep-cyclen and **(D)** NBD-Pep-cyclen. Scale bars: 100 nm. **(E)** CD spectrum of the nanofibers formed by LND-Pep-cyclen and NBD-Pep-cyclen. **(F)** Zeta potential values before and after cyclen modification at pH 6.5. **(G)** CLSM images of MCF-7 cells treated with NBD-Pep-cyclen nanofibers (50 μM) for 4 h, and subsequently stained with Mito-Tracker® Red probe and 4′,6-diamidino-2-phenylindole (DAPI). Scale bar for low magnification is 25 μm and for higher magnification is 15 μm, respectively.

The peptide of LND-GFFYK (LND-Pep) was straightly prepared according to solid phase peptide synthesis (SPPS), and was subsequently reacted with the active ester of tert-butyloxycarbonyl-protected cyclen, which was previously synthesized based on Figure S1. This finally upon de-protection yielded the product of LND-GFFYK-cyclen (LND-Pep-cyclen) (Figure S2). After high-performance liquid chromatography (HPLC) purification and freeze drying, the self-assembly performance of the obtained compound was verified. The results in Figure 1A showed that the peptide conjugate of LND-Pep-cyclen completely dissolved under heating, and assembled to form a macroscopically visible translucent hydrogel upon cooling. This is the first report of a supramolecular hydrogel formed by mitochondrion-targeted LND-peptide conjugate self-assembly without any organic solvent assistance, which can significantly improve the water-solubility and availability of LND. The nanostructure of the resultant hydrogel was observed via transmission electron microscopy (TEM), and it was found that the supramolecular “nano-boat” was actually composed of long and cross-linked nanofibers (Figure 1B). Furthermore, the spectra of circular dichroism (CD), as displayed in Figure 1E, demonstrated that the secondary structure of self-assembled nanofibers was β-sheet predominated, as the absorption spectrum presenting positive and negative peaks at the wavelengths of 190 nm and 210 nm, respectively. The zeta potential transition of peptide nanofibers caused by cyclen modification was measured. As shown in Figure 1F, the peptide nanofibers of LND-GFFYK (LND-Pep) possessed weak negative charged properties with a zeta potential of -7 mV. After cyclen modification, the surface zeta potential of LND-Pep-cyclen was elevated to above 40 mV at pH 6.5. It was noteworthy that, the zeta potential of LND-Pep-cyclen was decreased significantly with increasing pH, being only 17 mV at pH 7.0 and 8 mV at pH 7.4 (Figure S8), suggesting the protonation effect of cyclen under acid conditions. Therefore, the positive charged and the lipophilic properties most probably endow the LND-Pep-cyclen nanofibers with high potential to target the mitochondrion in cancer cells.

To confirm our hypothesis, we subsequently used a fluorescent molecule of 7-nitrobenzo-2-oxa-1,3-diazole (NBD), to replace LND, resulting in another peptide conjugate of NBD-GFFYK-cyclen (NBD-Pep-cyclen). The related results demonstrated that the NBD-Pep-cyclen could also self-assemble into a hydrogel upon heating-cooling (Figure 1C), and its internal morphology was also composed with long and thin nanofibers (Figure 1D). Moreover, it presented similar β-sheet predominated secondary structure (Figure 1E) and charge change properties (Figure 1F), comparable to those of LND-Pep-cyclen. Therefore, the NBD-Pep-cyclen conjugate could be very qualified as a substitute for LND-Pep-cyclen to verify its mitochondrial targeting performance. After incubation with MCF-7 cells for 4 hours, the cellular distribution of the NBD-Pep-cyclen nanofibers was investigated. As shown by the confocal laser scanning microscopy (CLSM) images in the Figure 1G, the green fluorescence of NBD was completely overlapped with the red fluorescence of the mitochondrial probe, proving that the designed supramolecular “nano-boat” could effectively target and locate at the mitochondrion of cancer cells. The endocytosis mechanism for the cellular uptake was investigated using several well-established endocytotic inhibitors. As shown in Figure S9, the addition of nystatin (an inhibitor of caveolae-mediated endocytosis) and wortmannin (an inhibitor of macropinocytosis) barely affected the uptake of NBD-Pep-cyclen nanofibers. In contrast, the addition of NaN_3_ (an inhibitor of energy-dependent endocytosis) and chlorpromazine (an inhibitor of clathrin-mediated endocytosis) reduced the uptake to less than 50%. These results indicated that the cellular uptake of the supramolecular “nano-boat” mainly underwent clathrin-mediated endocytosis and energy-dependent endocytosis.

### *In vitro* selective and enhanced cytotoxicity against cancer cells

**Figure 2:**
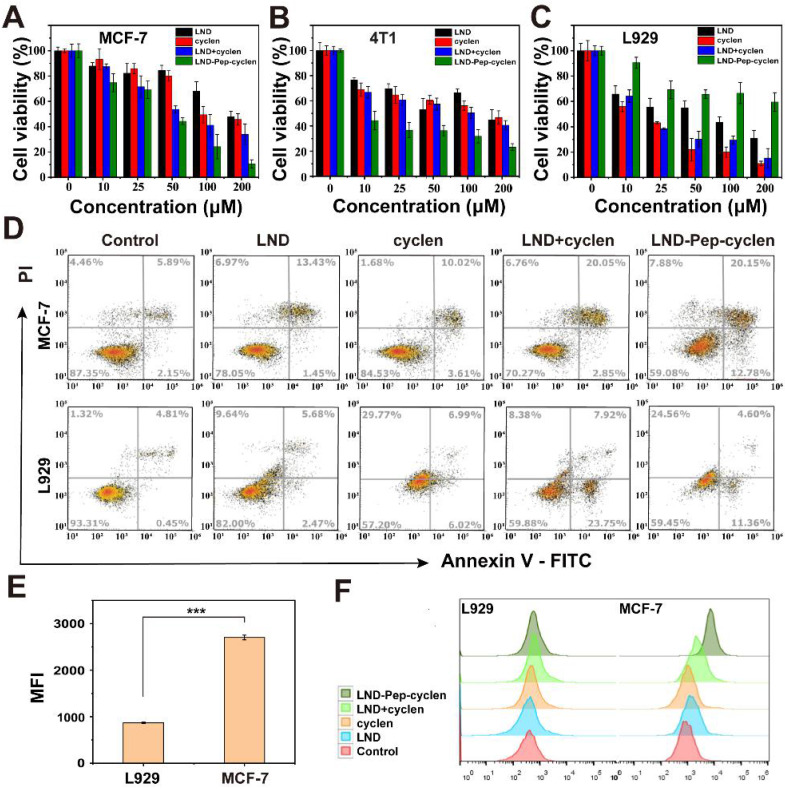
**Selective cytotoxicity of supramolecular “nano-boat” *in vitro*.** Cell viabilities of **(A)** MCF-7, **(B)** 4T1, and **(C)** L929 cells after 48 h of incubation with different formulations. The concentrations of both LND and cyclen were maintained to be the same in range of 0-200 μM. **(D)** Flow cytometry analysis of apoptosis cells after MCF-7 and L929 cells were treated with different formulations for 48 h at concentration of 50 μM. **(E)** Flow cytometry quantitative analysis of mean fluorescence intensity (MFI) of MCF-7 and L929 cells after treatment with NBD-Pep-cyclen nanofibers (50 μM) for 4 h (n = 3). ***P < 0.001. **(F)** Flow cytometry analysis of ROS generation of MCF-7 and L929 cells treated with indicated formulations (50 μM) for 12 h using DCFH-DA assay kit.

We evaluated the anti-proliferation effects of the free drug molecules and the self-assembled supramolecular “nano-boat” on different cells using the cell counting kit (CCK)-8 assay. The results in Figures 2A, 2B and S10 suggested that compared to free LND, the LND-loaded supramolecular “nano-boat” displayed much better inhibitory effect on the growth of cancer cells—including MCF-7, 4T1, HeLa, and A549 cells. For example, after co-incubating with MCF-7 cells for 48 hours, the cell survival rate of the group treated with free LND at 200 μM was still as high as 50%, whereas that of the group treated with the supramolecular “nano-boat” was reduced to less than 10% (Figure 2A). This enhanced anti-proliferation effect may be attributed to the increased uptake and subsequent mitochondrial location by the formation of bioactive nanofibers, leading to an improved bioavailability of LND in cancer cells. More importantly, when using the same method to evaluate the cytotoxicity of the different formulations on normal cells, it was found that the supramolecular “nano-boat” could apparently abate the cytotoxicity of free LND against L929 and HIEC-6 normal cells (Figure 2C and S10), which was diametrically opposite to the effect of the mixture of free LND and cyclen.

In addition, we further investigated the apoptosis-promoting abilities of the different formulations by flow cytometry. As shown in Figures 2D and S11, the apoptosis rate of the MCF-7 cells treated with the supramolecular “nano-boat” was elevated from 14.8% to 32.8% compared to the group treated with free LND. It was worth mentioning that the early apoptosis rate of the group was 12.78%, much higher than 1.45% of the free LND treated group, indicating that the tumor cells could effectively initiate a programmable death process under the stimulation of the supramolecular “nano-boat”. Meanwhile, the apoptosis rate of L929 cells induced by the supramolecular “nano-boat” was significantly lower than that of the mixture of free LND and cyclen. These results were basically consistent with the results of the CCK-8 experiments, indicating that the designed peptide “nano-boat” could specifically inhibit the growth of cancer cells and induce their apoptosis.

We conducted a flow cytometry quantitative analysis of the NBD-loaded supramolecular “nano-boat” to compare their accumulation in cancer cells and normal cells. As shown in Figures 2E and S12, the supramolecular “nano-boat” presented a much higher retention in MCF-7 cells than that in L929 cells, manifested by the fluorescence intensity of NBD in the former was being more than two-folds than that in the latter. This is most probably likely caused by the higher MMP of the cancer cells, which caused the internalized supramolecular “nano-boat” to more easily sail to and locate at the mitochondrion. Moreover, a flow cytometry quantitative analysis of the reactive oxygen species (ROS) generation with different treatments was also performed. The results in Figures 2F, S13 and S14 suggested the supramolecular “nano-boat” could significantly boost the production of ROS in MCF-7 cells, whereas it barely promoted that in L929 cells compared to the effect of the free drug. It is well known that mitochondria are the main provenance of excessive cellular ROS upon exogenous stimulation 49. Hence, the large ROS in cancer cells probably originated from the mitochondrial impairment and dysfunction caused by the supramolecular “nano-boat”. The elevated oxidation pressure, in turn, further damaged mitochondrion, which eventually forming a vicious circle and inducing cancer cell apoptosis. Therefore, the peptide “nano-boat” hold significant potential for achieving selective anti-tumor therapy, and simultaneously reducing the cytotoxicity of anticancer drugs on normal cells.

### MMP depolarization and apoptotic proteins expression

**Figure 3:**
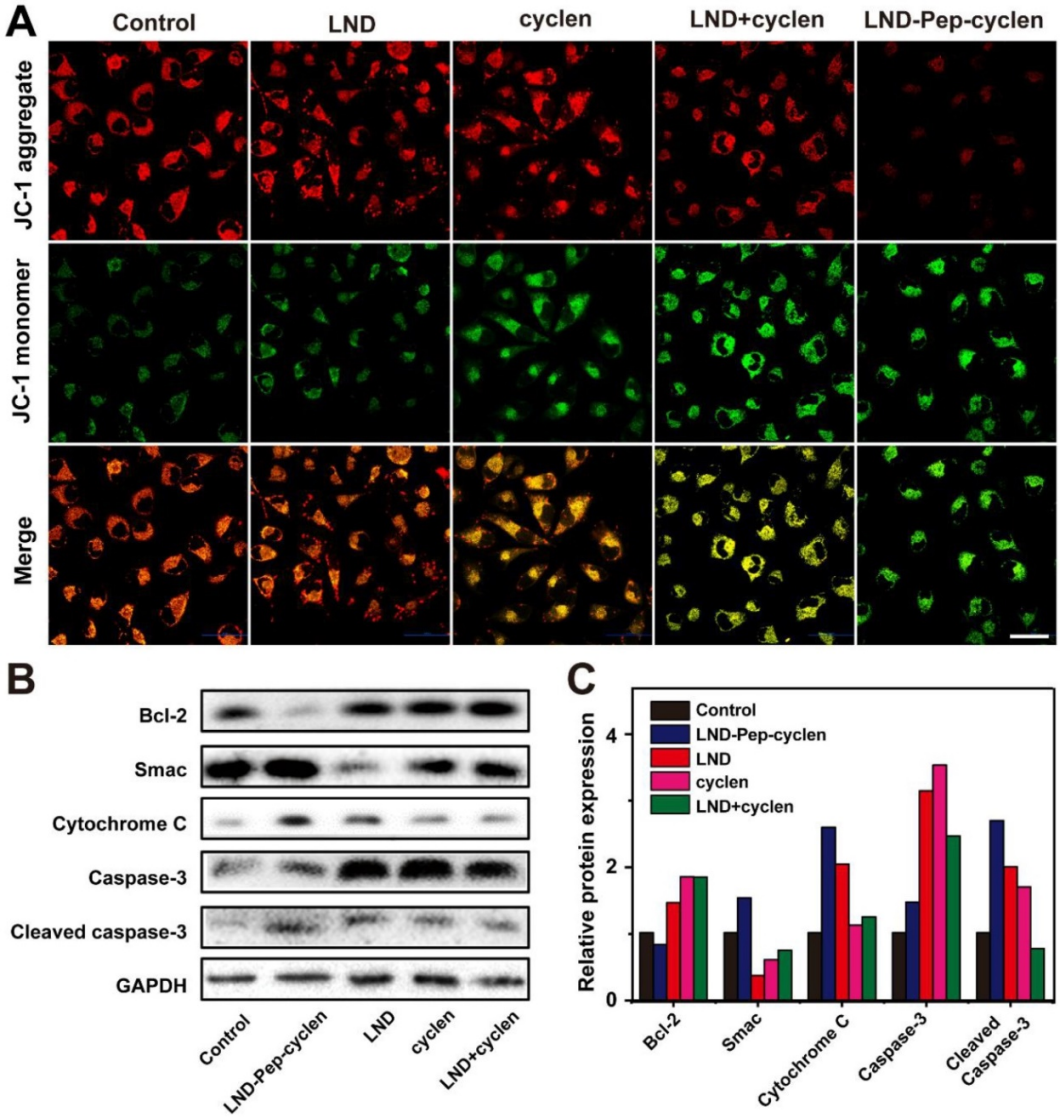
**MMP depolarization and apoptosis-related proteins expression. (A)** CLSM images of MCF-7 cells treated with different formulations (50 μM) for 24 h, and subsequently stained with JC-1 probe for 30 min. Scale bar: 50 μm. **(B)** Western blot images and **(C)** Quantitative analysis of Bcl-2, Smac, cytochrome C, caspase-3, and cleaved caspase-3 levels in the cell lysate after MCF-7 cells were incubated with different treatments (50 μM) for 48 h.

Normal MMP is a precondition for maintaining the mitochondrial energetic source, and is conducive for the normal cellular physiological function 50-52. To further prove the mitochondrial dysfunction of the MCF-7 cells treated with the supramolecular “nano-boat”, we observed the MMP changes before and after different treatments. After incubating with MCF-7 cells for 24 hours, a JC-1 probe was employed to monitor the depolarization of the different treatments on the MMP. It has been known that in normal mitochondrion, JC-1 aggregates in the mitochondrial matrix to form polymers emitting a strong red fluorescence, whereas in an unhealthy mitochondrion, it can only exist in the cytoplasm in the form of monomers, emitting a green fluorescence 53. As shown in Figure 3A, a strong red fluorescence was seen in both the control cells and the free LND treated cells, indicating the very limited effect of free LND on the MMP depolarization. Conversely, the red fluorescence basically disappeared and was replaced by a strong green fluorescence in the cells treated with LND-Pep-cyclen, indicating that the MMP is almost completely dissipated. This result was in good agreement with Figure 2D, as the destruction of MMP is a landmark event in the early stage of cell apoptosis.

Moreover, we analyzed the expression levels of apoptosis-related proteins to verify whether the LND-Pep-cyclen triggering the endogenous mitochondrial apoptosis pathway. The results in Figures 3B and 3C indicated that compared to the control group and free drug groups, the anti-apoptosis protein level of Bcl-2 in the group treated with LND-Pep-cyclen was significantly down-regulated. In contrast, the apoptosis initiation proteins including Smac and cytochrome C were both significantly up-regulated. Notably, the expression level of cytochrome C was particularly high in the cells treated with LND-Pep-cyclen, whereas it was barely detectable in the other groups, suggesting that LND-Pep-cyclen could more effectively to damage the mitochondrial membrane to trigger abundant of cytochrome C leakage. In addition, caspase-3 in the cells treated with LND-Pep-cyclen also underwent more prominent degradation than in the other treatments, which eventually caused a relative high expression of active cleaved caspase-3 to induce cell apoptosis. The above results indicated that the peptide “nano-boat” could effectively induce mitochondrial dysfunction and activate the mitochondrial apoptosis pathway to finally kill the cancer cells.

### OXPHOS inhibition and glycolysis inhibition

**Figure 4:**
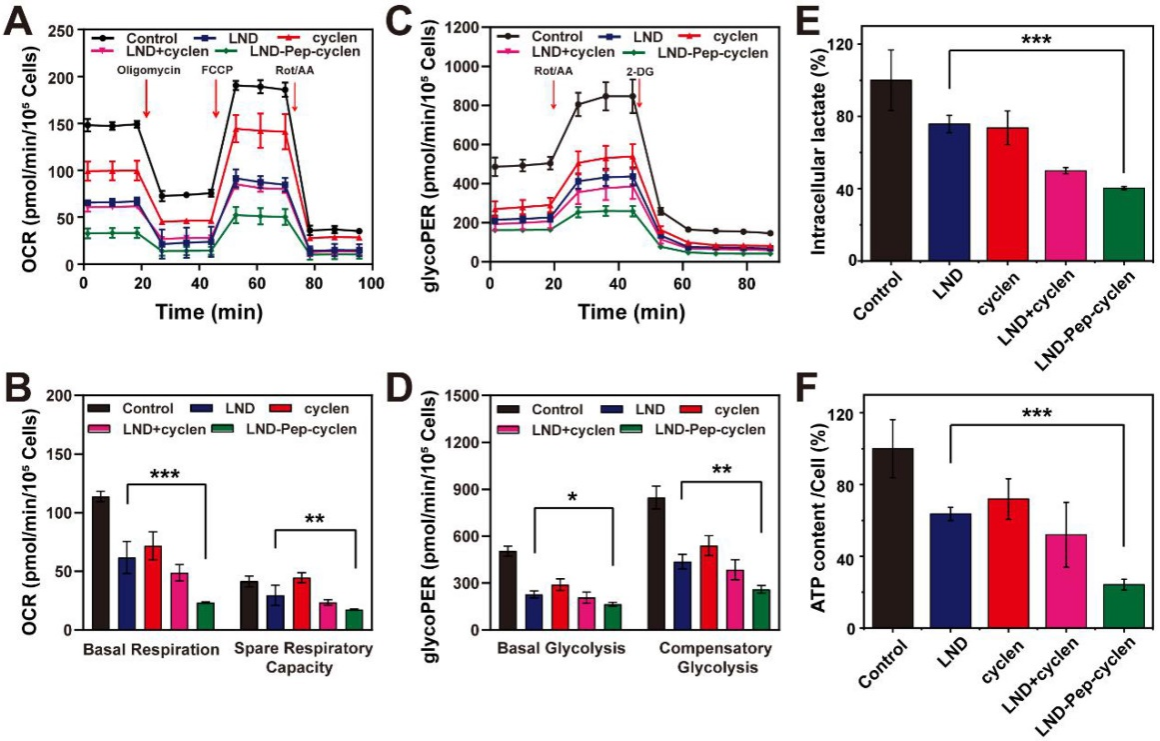
** OCR and glycolysis inhibition.** Kinetic profiles of OCR** (A)** and glycoPER **(C)** in MCF-7 cells treated with different formulations (50 μM) for 12 h. **(B)** Quantification of basal respiration and spare respiratory capacity from the kinetic profiles of OCR. **(D)** Quantification of basal glycolysis and compensatory glycolysis from the kinetic profiles of glycoPER. **(E)** Intraceller lactate and **(F)** ATP content in per cell after MCF-7 cells were treated with different formulations (50 μM) for 24 h. The values of the control group were all set as 100%, and the data was shown as the mean ± SD (n = 5). *P < 0.05, **P < 0.01, ***P < 0.001.

We tested the oxygen consumption rate (OCR) and glycolytic proton efflux rate (glycoPER) of MCF-7 cells after different treatments using a Seahorse XFe24 energy analysis system. As shown in Figure 4A, the OCR and of the pre-treated cells was inhibited to different agrees compared to that of the un-treated control group. Apparently, the cells treated with LND-Pep-cyclen exhibited the lowest OCR value during the entire measurement. The basal respiration and the spare respiratory capacity were further quantified. As shown in Figure 4B, LND-Pep-cyclen induced an approximately 80% decrease in the basal respiration, which was more than two folds than that by free LND. Moreover, the spare respiratory capacity of the LND-Pep-cyclen treated cells was much lower than that of the free LND treated cells, indicating the adaptability of the cells to respond external threats became much worse. The glycoPER was also performed to examine the glycolytic capacities of the cells after different treatments. As shown in Figure 4C, the LND-Pep-cyclen treated cells displayed the lowest glycoPER value among all groups. Both the basal glycolysis and compensatory glycolysis of the LND-Pep-cyclen treated cells were significantly inhibited, which possessing statistical differences in comparison with those of the free LND treated cells (Figure 4D). The OCR versus extracellular acidification rate (ECAR) profile in the Figure S15 more objectively reflect that the two major energy production pathways were significantly inhibited by the supramolecular “nano-boat”, as this led to drastic decreases in both OCR and ECAR compared to those in the other treatments.

On the other hand, as the end product of glycolysis, lactate also can be regarded as another indicator for verifying whether glycolysis inhibition. Specifically, the glycolysis inhibition is typically accompanied with the reduction in lactate 10. The result in Figure 4E demonstrated that as compared with the free drugs, the LND-Pep-cyclen was more effective in decreasing the intracellular lactate concentration, which was coincident with the result in Figure 4C. It is well known that the first step in glycolysis is the glucose being catalyzed by HK to 6-phosphate glucose. Subsequently, the activity of HK-II in the MCF-7 cells of each group was tested to further investigate the mechanism of glycolysis inhibition. As shown in Figure S16, all the treatments involving free LND could cause HK-II to deactivate to different extents. Particularly, the activity of HK-II in the cells treated with LND-Pep-cyclen decreased to less than 40% of that in the control group, which was only one half of that in the free LND treated cells. Furthermore, the ATP contents of the MCF-7 cells after different treatments were quantitatively recorded. As seen in Figure 4F, the ATP content in the cells treated with LND-Pep-cyclen was only 20% of control, which was much lower than the 60% of the control in the free LND treated cells, further indicating the powerful energy metabolism inhibition capacity of LND-Pep-cyclen. Therefore, all the above results fully confirmed that LND-Pep-cyclen was competent to block the energy metabolism of cancer cells by a combination of glycolysis and oxidative phosphorylation inhibition.

### Mitochondrial ultrastructure change induced by the supramolecular “nano-boat”

**Figure 5:**
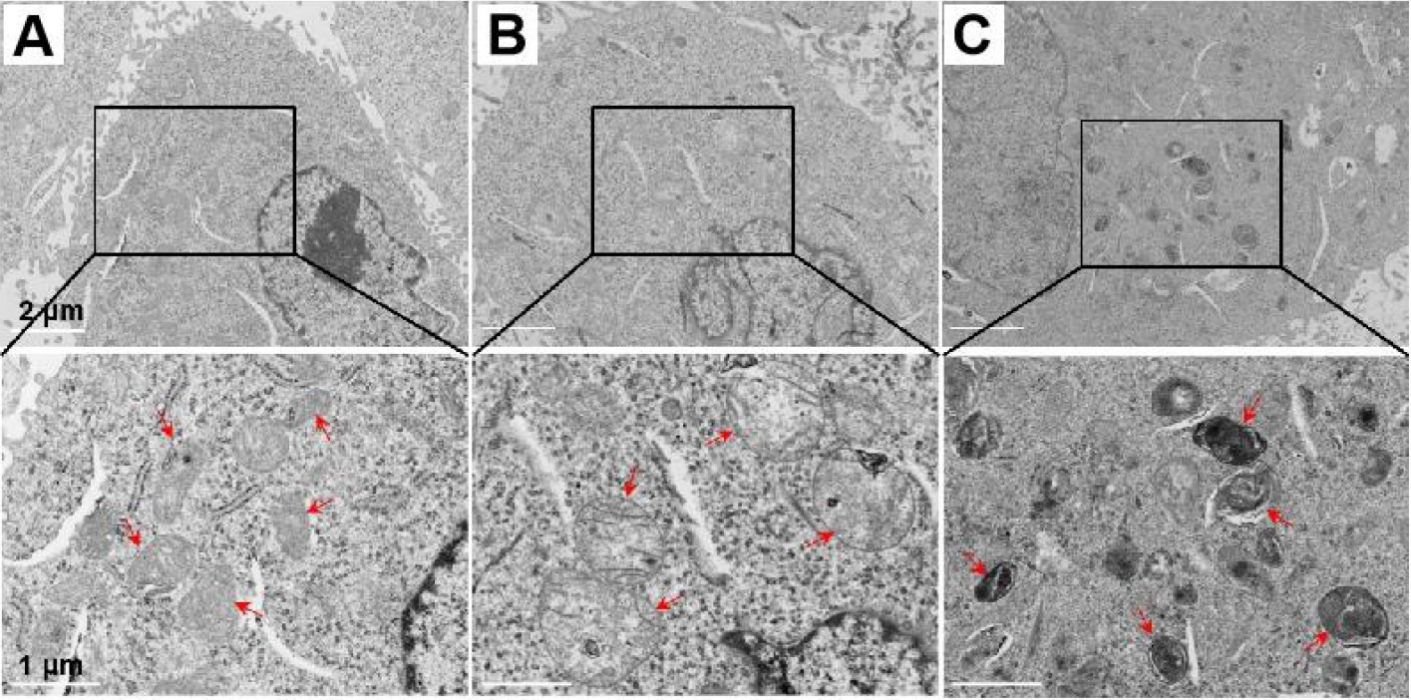
**Representative Bio-TEM images of mitochondrial ultrastructure.** The mitochondrial ultrastructure change in MCF-7 cells before **(A)** and after treatment with LND-Pep-cyclen nanofibers (50 μM) for 12 h **(B)** and 24 h **(C)**. Red arrows indicated representative mitochondrion.

Mitochondrial respiration is frequently closely determined by the mitochondrial morphology. The remarkable OCR decrease reflected severe damage of the mitochondrial electronic respiratory chain, probably due to the ultrastructure changes of the mitochondrial morphology after the LND-Pep-cyclen treatment. To prove this, we observed the morphological changes of the mitochondrial ultrastructure by biological transmission electron microscopy (Bio-TEM) before and after LND-Pep-cyclen treatment. As shown in Figure 5A, in the normal MCF-7 cells of the control group, most of the mitochondria were oval and their intima folded into noticeable cristaes. After incubating with LND-Pep-cyclen for 12 hours, the mitochondria in the cells obviously swelled causing enlarged volume, and the mitochondrial cristaes basically disappeared simultaneously (Figure 5B). When prolonging the incubation time to 24 hours, the mitochondrial pyknosis occurred in the cells treated with LND-Pep-cyclen, resulting in increased mitochondrial electron density and a marked deepening staining (Figure 5C). The ultrastructure changing one more time confirmed the LND-Pep-cyclen could exactly destroy the mitochondrial function in the cancer cells. Based on the above results, the designed supramolecular “nano-boat” could disrupt the morphology of the mitochondrion, depolarize the MMP, and subsequently induce cancer cells apoptosis.

### *In vitro* radio-sensitization effect of the supramolecular “nano-boat”

**Figure 6:**
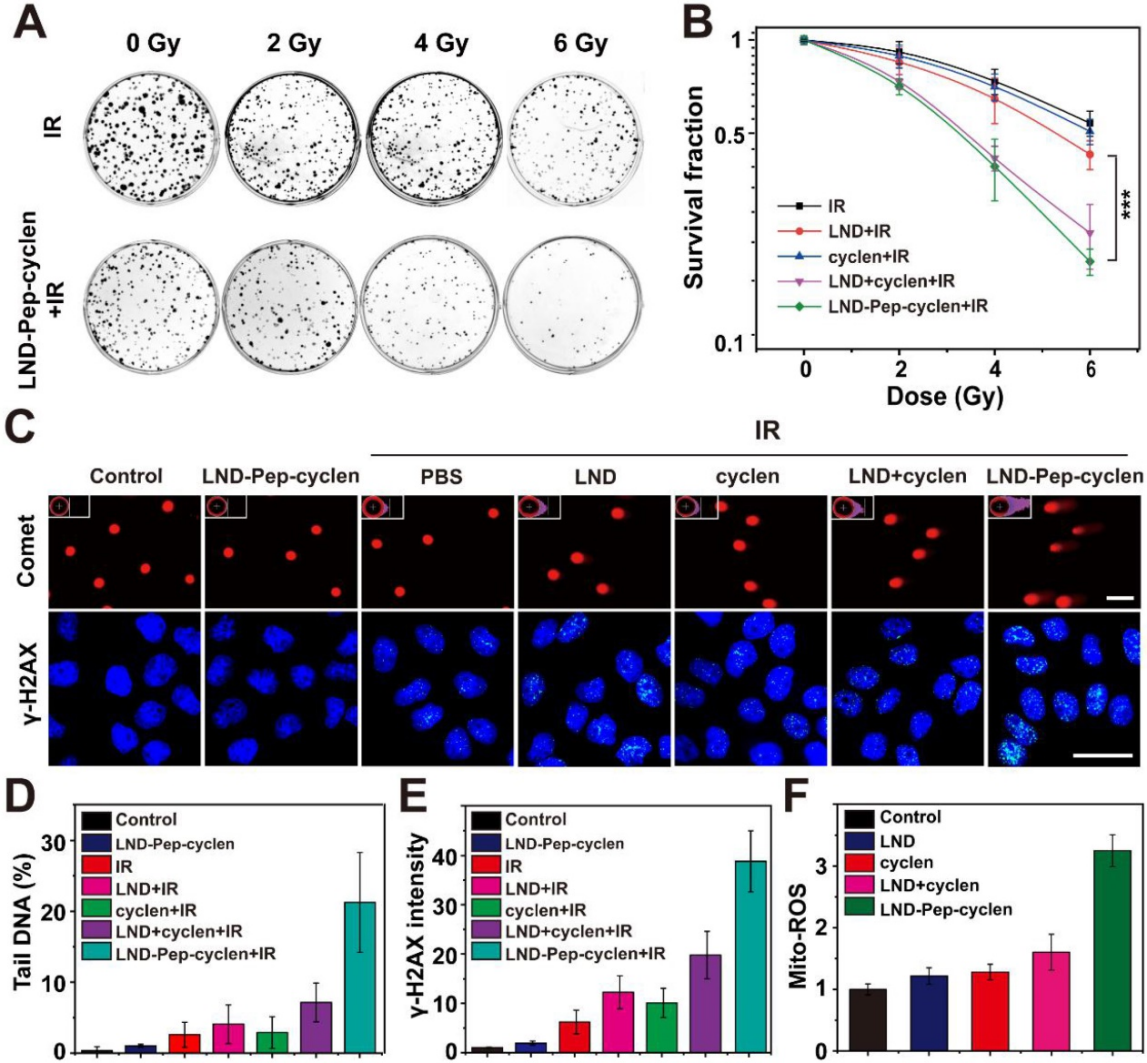
*** In vitro* radio-sensitization effect of supramolecular “nano-boat”. (A)** Colony formation photographs of MCF-7 cells treated with the indicated formulations (10 μM) for 12 h, and subsequently exposed to γ-rays with different doses. **(B)** Colony formation survival curves of MCF-7 cells treated with different formulations and irradiation doses. Data were shown as the mean ± SD (n = 3). ***P < 0.001. **(C)** Comet assay images of DNA fragmentation, and immunofluorescence images of γ-H2AX foci in MCF-7 cells (nuclear was shown in blue whereas γ-H2AX in green). scale bars: 25 μm. **(D)** Quantitative analysis of tail DNA content in comet assay using CASP software. **(E)** Fluorescence intensity of γ-H2AX foci in each group were measured using Image J software, and the obtained value in the control group was set as 1. **(F)** Flow cytometry quantitative analysis of Mito-ROS production in MCF-7 cells treated with indicated formulations (10 μM) for 12 h using mitochondrial superoxide indicator of MitoSOX™ Red probe. Data were shown as the mean ± SD (n = 3).

The capability of LND-Pep-cyclen to decrease oxygen consumption endowed it with high potential as a radio-sensitizer to enhance the therapeutic effects of radiotherapy, because radiotherapy resistance is frequently associated with tumor hypoxia 53. Therefore, we evaluated the radio-sensitization effects of different formulations on MCF-7 cells *in vitro* at a non-toxic concentration. As shown in Figure S17, after incubation with the different drugs at such a lower concentration for 12 h, the cell viability of the MCF-7 cells in each group was still up to 100%. Subsequently, the clone formation assay of MCF-7 cells after different treatments and different radiation doses was conducted. The results in Figures 6A and S18 indicated that the free LND had very limited inhibition ability for colony formation compared with only ionizing radiation (IR), even when the radiation dose ranged from 2 Gy to 6 Gy. In contrast, the LND-Pep-cyclen displayed prominent inhibition ability for colony formation, particularly at higher radiation doses of 4 Gy and 6 Gy. Moreover, the survival fraction curves in Figure 6B demonstrated that there was a significant statistical difference between free LND and LND-Pep-cyclen. As calculated from the curves, the SER value of LND-Pep-cyclen was determined as 2.58, which was much larger than that of free LND (1.78), suggesting a dramatic enhanced radio-sensitization effect when LND was fabricated into mitochondria-targeted supramolecular “nano-boat”.

Subsequently, we performed the comet assay to analyze the DNA damage of a single cell. A long length of the tail of a cell implies severe DNA damage. As shown in Figure 6C, after LND-Pep-cyclen treatment without IR, the cells almost had no tails similar to those in the control group. On being irradiated under 6 Gy, the cells treated with LND-Pep-cyclen possessed the longest tails and the largest fluorescence area among those in all groups. As quantitated in Figure 6D, the content of the tail DNA in the cells treated with LND-Pep-cyclen and IR was more than 20%, much higher than that in free LND and IR treated cells. The γ-H2AX staining was used to further detect the double-strand breaks (DSBs) of DNA in the cells of different groups. The results in Figure 6C showed that different numbers of green fluorescent spots appeared in the nuclei of the irradiated cells, indicating that the irradiation caused DNA damage by various degrees. By the quantitative comparison in Figure 6E, it was founded that the most DSBs were occured within the nuclei of the cells treated with LND-Pep-cyclen, being as high as approximately four-folds more than that in free LND treated cells. The above results strongly proved that the LND-Pep-cyclen could be viewed as an effective radio-sensitizer for enhancing radiotherapy efficiency.

The enhanced radio-sensitization effect of LND-Pep-cyclen could be attributed its destruction of the mitochondrial respiratory chains, which could cause superoxide anion leakage, resulting an elevated mitochondrial superoxide (Mito-ROS). Therefore, we qualitatively and quantitatively analyzed the content of Mito-ROS in cells treated with different materials. The results in Figures 6F and S19 showed that among all groups, the cells treated with LND-Pep-cyclen exhibited the strongest red fluorescence of the MitoSOX™ Red probe, with the amount of Mito-ROS being at least twice of those in the other groups. The enhanced oxidative stress combined with radiation could effectively cause the apoptosis via DSBs and the activation of tumor suppressor p53 54, finally inducing the radio-sensitization effect of the supramolecular “nano-boat” against the cancer cells. Therefore, the LND-Pep-cyclen was endowed with dual functions of both chemotherapy and radio-sensitization, making it highly promising to achieve simultaneous chemo-radiotherapy and excellent anticancer effect.

### *In vivo* synergistic chemo-radiotherapy anti-tumor evaluation

**Figure 7:**
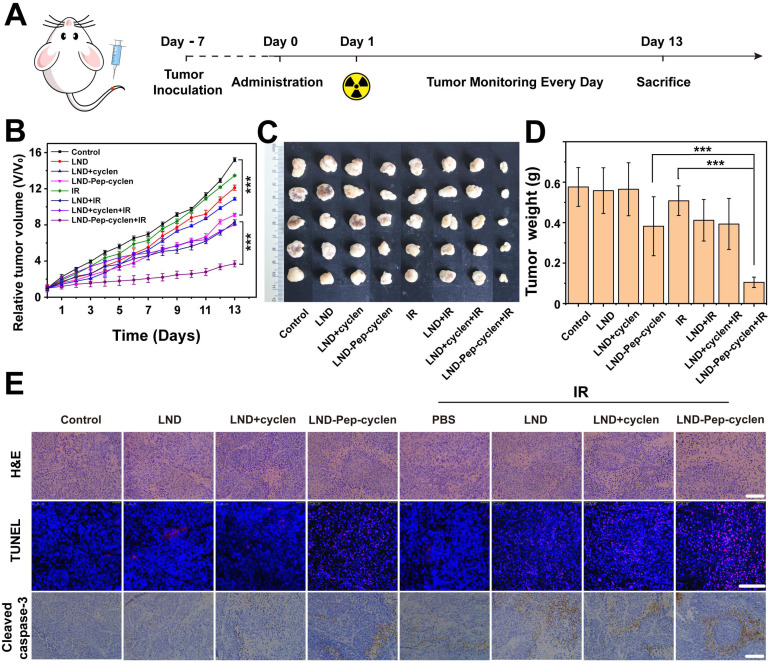
***In vivo* synergistic chemo-radiotherapy anti-tumor effect of supramolecular “nano-boat” on MCF-7 tumor bearing nude mice. (A)** Schematic illustration of the *in vivo* therapy protocol. **(B)** Tumor growth curves, **(C)** Tumor images, and **(D)** Tumor weight in each group after treatment with different formulations with or without 6 Gy dose of γ-ray irradiation. Data were shown as the mean ± SD (n = 6). ***P < 0.001. **(E)** H&E staining, TUNEL and cleaved caspase-3 staining for the tumor tissues in each group. Scale bars: 100 μm.

We evaluated the *in vivo* anti-tumor effects of only chemotherapy and combined chemo-radiotherapy by establishing subcutaneous xenografted nude mice model bearing MCF-7 tumors. As illustrated in Figure 7A, different formulations were administrated by tail vein injections in all the groups, and 12 h later, 8 Gy of radiation dosage was administered at the tumor sites of the chemo-radiotherapy groups. The therapeutic efficiency was firstly evaluated through tracking the tumor volume changes of the different groups. As seen in Figure 7B, the free LND based chemotherapy could barely inhibit the tumor growth, as presented by the final tumor volumes of tumors being very close to that of the phosphate buffer saline (PBS) control group. The unremarkable anti-tumor effect was due to the poor tumor accumulation capacity and rapid excretion *in vivo* of the free drug. Interestingly, the LND-Pep-cyclen with single injection apparently improved the anti-tumor effect, illustrated by the final tumor volume being only one half of that of the PBS control group. The EPR effect and mitochondrion-targeting ability of LND-Pep-cyclen could be seen as the main key factors causing the enhanced tumor suppression effect. Even more promisingly, in the LND-Pep-cyclen treated group, the tumor growth was further suppressed once combined with radiotherapy, and the final tumor volume was reduced to as low as less than one half of that with only chemotherapy. Concurrently, no notable body weight loss was observed during all the treatments (Figure S20), suggesting low systemic toxicity of single chemotherapy and radiotherapy.

In addition, the photos of exfoliated tumors shown in Figure 7C had provided a highly objective basis for evaluating the anticancer effects of the different treatments. By weighing the isolated tumors, it was found that the tumor weight of the combined chemo-radiotherapy group was only 0.1g, which was much lower than 0.38 g of the chemotherapy group and 0.42 g of the radiotherapy group (Figure 7D). Accordingly, the tumor inhibition rate ( Figure S21) treated with chemo-radiotherapy treatment was up to 80%, far higher than the sum of the rates with chemotherapy alone (33%) and radiotherapy alone (10%), suggesting a synergetic antitumor effect of 1 + 1 > 2. Meanwhile, the immunohistochemical analyses, including H&E, TUNEL, and caspase-3 expression were further performed to examine the anti-proliferation and apoptosis levels in tumor tissues. As shown in the H&E images in Figure 7E, the tumor cell growth was the most severely inhibited in the LND-Pep-cyclen treated group with radiotherapy, as displayed by the number of nuclei being remarkably decreased comapred to those in the other groups. At the same time, the largest numbers of red fluorescent spots were observed in the TUNEL images of the LND-Pep-cyclen mediated chemo-radiotherapy group, which indicated the most severe DNA breakage. In addition, the expression level of apoptosis-related protein caspase-3 in this group was much higher than those in the other groups. These results fully validate vindicated that mitochondrion-targeted chemo-radiotherapy can* in vivo* effectively inhibit tumor growth and promote tumor cell apoptosis.

### *In vivo* bio-compatibility assessment of the supramolecular “nano-boat''

**Figure 8:**
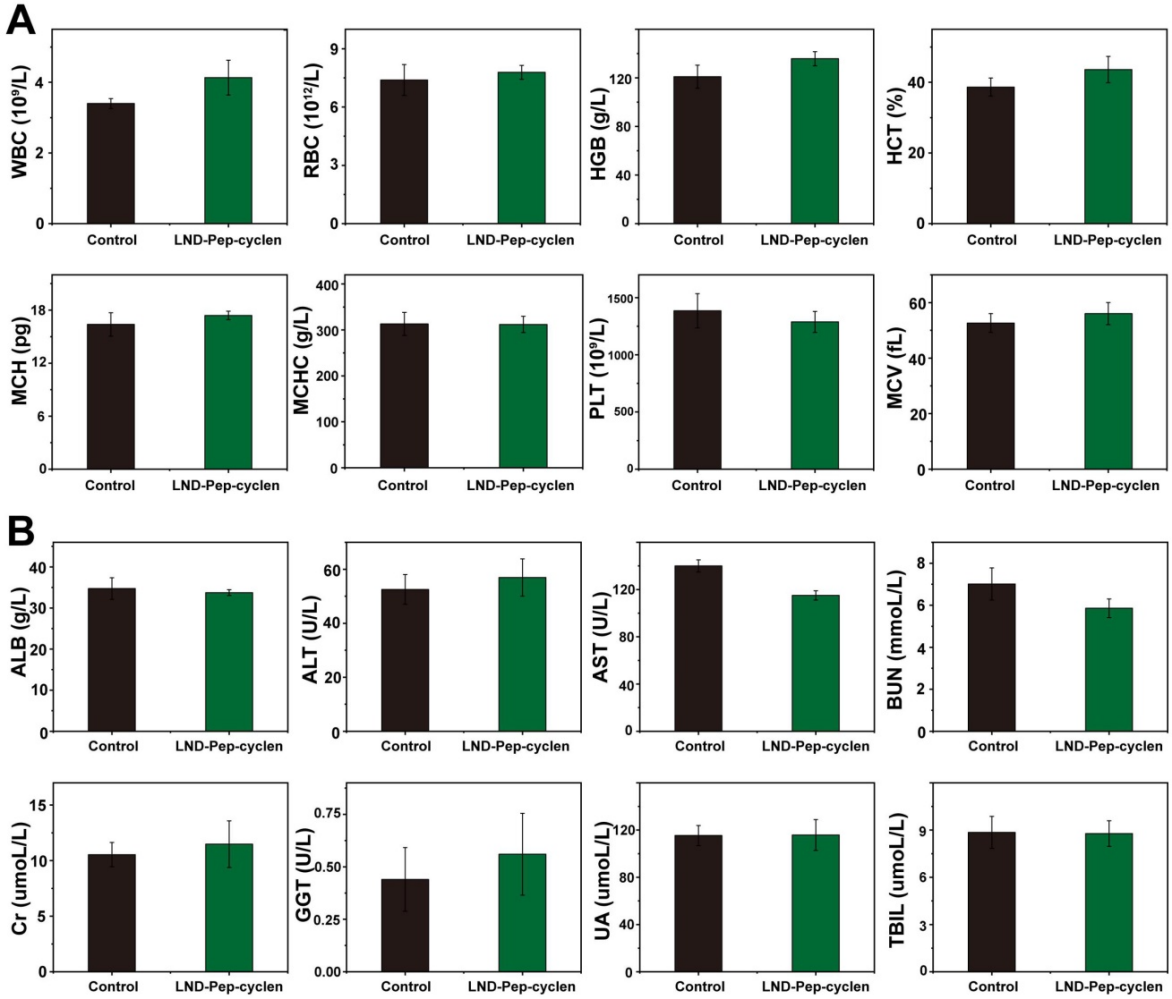
***In vivo* bio-compatibility assessment of supramolecular “nano-boat”. (A)** Hematology results of different treatments. **(B)** Blood biochemical results of different treatments. All data were shown as mean ± SD (n = 5).

The histological changes in the major organs including the liver, spleen, kidney, heart, and lung were examined after different treatments by H&E immunohistochemistry. As shown in Figure S22, no appreciable inflammatory damage and tissue damage was observed in the supramolecular “nano-boat” treated group compared with those in the control group, suggesting single administration possessed good safety. The potential *in vivo* systematical toxicity of the designed supramolecular “nano-boat” was further evaluated by blood routine and serum biochemistry assay. As shown in Figure 8A, after four times injections, there were no apparent variations in the blood routine values between the supramolecular “nano-boat” treated group and the untreated control group, corroborating that the supramolecular “nano-boat” do not produce notable deleterious effects. In addition, the main indices of liver and kidney, such as alanine transaminase (ALT), aspartate transaminase (AST), total bilirubin (TBIL), and blood urea nitrogen (BUN) are evaluated as well. As seen in Figure 8B, almost all the results obtained from the supramolecular “nano-boat” treated group were normal with no relevant difference as compared to those of the control group. All above results indicated that the designed supramolecular “nano-boat”, as an innovative mitochondria-targeted drug delivery nano-platform, possessed excellent biocompatibility and major potential for possible further clinical applications.

## Conclusions

In summary, we have constructed a novel mitochondrion-targeted self-delivery system based on peptide self-assembly (which could be visually called a supramolecular “nano-boat") for cancer chemo-radiotherapy. The supramolecular “nano-boat” could significantly enhance the anti-proliferation effect of free LND against tumor cells, and simultaneously reduce its cytotoxicity against normal cells. The selective anti-tumor effect of the supramolecular “nano-boat” was mainly attributed to its much higher accumulation in cancer cells than in normal cells. The mitochondrial ultrastructure in the cancer cells was severely damaged by the supramolecular “nano-boat”, leading to the depolarization of MMP, abundant production of ROS and increased expression of mitochondrial apoptotic proteins. Meanwhile, both the dual energy pathways of glycolysis and oxidative phosphorylation were effectively inhibited in the supramolecular “nano-boat” treated cancer cells, manifested as the reduced intracellular lactate, oxygen consumption rate, and ATP content. In addition, the prominent radio-sensitization effect of the supramolecular “nano-boat” *in vitro* was fully confirmed, which finally produced a synergistic anti-tumor effect when combined with radiotherapy, leading to the tumor inhibition rate of a single chemotherapy plus radiotherapy* in vivo* of as high as 80%. Considering the inherent advantages of the peptide carrier, such as flexible design, low-cost and good biocompatibility, the supramolecular “nano-boat” hold high potential for future clinical translations and applications. This research not only expanded the feasibility of peptide-based nanomedicine in cancer chemo-radiotherapy, but also provided a universal strategy for designing novel mitochondrion-targeted drug delivery systems for cancer-specific therapy.

## Materials and Methods

### Materials

Fmoc-amino acids, O-Benzotriazole-N, N, N', N'-tetramethyl-uronium-hexafluorophosphate (HBTU), and 2-Cl-trityl chloride resin were purchased from GL Biochem (Shanghai, China). Lonidamine, 4-chloro-7-nitrobenzo-2-oxa-1,3-diazole, N-hydroxysuccinimide, cyclen, fluorescein isothiocyanate, trifluoroacetic acid (TFA), diisopropylethylamine (DIEA), and triisopropylsilane (TIS) were purchased from J&K Chemical Technology (Beijing, China). Cell culture media, penicillin, and streptomycin, fetal bovine serum (FBS), and trypsin-ethylenediamine tetraacetic acid (EDTA) (0.25%) were obtained from Gibco. FITC Annexin-V apoptosis detection kits were obtained from BD Bioscience Pharmingen. Ultrapurified water was obtained from a Milli Q Plus system. Phosphate buffer saline (PBS) was obtained from Solarbio (Beijing, China). The CCK-8, ROS assay kit (DCFH-DA), and JC-1 probe were purchased from Yeasen Biotechnology (Shanghai, China).

### Preparation of peptide nanofibers (supramolecular “nano-boat”)

The peptide nanofibers of LND-Pep-cyclen and NBD-Pep-cyclen used in the following experiments were obtained by directly diluting and breaking up their corresponding hydrogels using double distilled water (ddH_2_O), PBS, or dulbecco's modified eagle medium (DMEM). The hydrogel of LND-Pep-cyclen was prepared by a heating-cooling method. Typically, LND-Pep-cyclen (4 mg) was first dissolved in 1 mL of ddH_2_O, to which subsequently Na_2_CO_3_ was added to adjust the final pH to 7.0. A bottle containing the solution was heated on an alcohol lamp to completely dissolve the peptide, and subsequently it was kept without moving until the solution cooled down and formed a hydrogel. The hydrogel of NBD-Pep-cyclen was prepared using the same procedures.

### Mitochondrial co-localization imaging assay

MCF-7 cancer cells with a density of 1×10^5^ cells/mL were cultured in confocal cuvettes at 37 °C for 4 h. After the culture medium was removed, the NBD-Pep-cyclen nanofibers (50 μM) in an FBS-free cell culture medium were subsequently added into each chamber. After 4 h of incubation, the cells were stained with MitoTracker® Red probe (100 nM) for 15 min followed by fixation using 4% paraformaldehyde. After being further incubated with DAPI (10 μg/mL) for 30 min, fluorescent images were captured by CLSM (Nikon eclipse Ti2). DAPI: *e_x_*=408 nm,* e_m_*=460 nm; NBD: *e_x_*=488 nm,* e_m_*=550 nm; Mito-Tracker:* e_x_*=579 nm,* e_m_*=610 nm.

### MMP (Δψm) depolarization

MCF-7 cancer cells with a density of 2 × 10^5^ cells/mL were cultured in confocal cuvettes under 5% CO_2_ at 37 °C. After the number of anchorage-dependent cells reached up to 70%, the cells were incubated with a replaceable serum-free medium containing different forms of the drug (50 μM) for 24 h. This was followed by washing away the drug that was not absorbed and staining with 1 mL JC-1 probe (10 µg/mL) for 30 min in dark. After washing with PBS for three times, the cells were imaged by CLSM to examine the fluorescence intensity of the J-monomers and the J-aggregates.

### Mitochondrial Bio-TEM morphology observation

A total of 1×10^6^ MCF-7 cells were plated in 10-cm dishes and then cultured in an incubator for 18 h. After treatment with LND-Pep-cyclen (50 μM) nanofibers for 0 h, 12 h, and 24 h and washing several times with PBS, the harvested pellets were sequentially fixed with 2.5% glutaraldehyde and 1% OsO_4_ at 4 °C. Subsequently, the fixed samples were dehydrated sequentially using solutions of ethyl alcohol (50%, 75%, 90% and 100%) and acetone (100%) prior to embedding over a period of 24 h with increased concentrations of a resin in acetone (25%, 50%, 75% and 100%). Following this, cell slices of different groups were obtained in an ultramicrotome after a series of programmed treatments, which were then subsequently mounted onto copper grids, followed by staining with 2% uranyl acetate and lead citrate solution. The mitochondrial morphology in the different samples was observed via TEM.

### OCR and glycoPER measurement

The OCR and the glycoPER were measured using a Seahorse instrument (XF24, Agilent). Briefly, the sensor cartridge was hydrated a day before the test, and the MCF-7 cells were inoculated into a specific culture plate for overnight culture. After treatment with different compounds for 12 h, the cells were changed into the Seahorse detection liquid and incubated at 37 °C for 60 min without CO_2_ supply. The OCR was measured under basal conditions and with the addition of oligomycin (1 μM, introduced after 28 min), carbonyl cyanide p-trifluoromethoxy phenylhydrazone (FCCP, 0.5 μM, introduced after 44 min), and rotenone/antimycin A (Rot/AA, 0.5 μM, introduced after 80 min). After the same pre-treatment, the glycoPER was measured under basal conditions and with the addition of Rot/AA (0.5 μM, introduced after 20 min) and 2-deoxy-D-glucose (2-DG, 0.5 μM, introduced after 58 min). Finally, both the results of OCR and glycoPER were obtained by data normalization processing. The base respiration was equal to the difference of the initial and termination values of the OCR, and the spare respiratory capacity was equal to the difference of the maximum and initial values of the OCR. The basal glycolysis and the compensatory glycolysis were equal to the initial and the maximum values of the glycoPER, respectively.

### Measurement of hexokinase (HK) activity, lactate content and ATP content

MCF-7 cells with a density of 5000 cells/well were seeded in 96-well plates, and then were cultured at the incubators for 24 h at 37 °C under 5% CO_2_. After co-incubation with different forms of drugs for another 24 h, the growth medium was removed, and the wells were washed twice with PBS. The cell number of each group was counted before all the measurements. After dissociation and centrifugation, the liquid supernatant was collected for the following analysis of the HK activity and the lactate content. The hexokinase (HK) activity of the cells in each group was determined by the OD_340_ absorbance according to the specification of the HK activity assay kit (Solarbio, BC0745). The intracellular lactate content was detected using an L-lactate assay kit (Promega, J5021) and depended on the OD_565_ absorbance. A luminescent ATP detection assay kit (Abcam, ab113849) was used to analyze the quantity of ATP. All the data were shown as the mean value in per cell of each group.

### Colony formation assay

MCF-7 cells (1,000/well) were seeded in six-well plates and incubated with different forms of drugs at a concentration of 10 μM after 12 h. Subsequently, they were irradiated with γ-rays at doses of 0, 2, 4, and 6 Gy at 36 h. Following this, the cells were continuously cultured with a fresh drug-free medium in an incubator for another 5-8 days. The fixed colonies were then stained using crystal violet (0.25%, ethanol) for subsequently counting the number of colonies, and evaluating the colony inhibition ability of each treatment. Only colonies containing >50 cells were counted and survival curves were plotted with Origin. Furthermore, the SER value was used to compare the efficiencies of the different drugs in improving the sensitivity of the MCF-7 cells to radiotherapy. The SER was determined by a classical multi-target single-hit model using the following equation:

*S* = 1 - (1 - e ^-*D*/*D0*^)*^n^* (1)

where* S* is the survival fraction, *D* is the radiation dose, *D_0_* is the mean lethal dose, and *n* is the extrapolation number. *D_0_* and *n* were calculated using the“GraphPad Prism” software (version 8.0). The final survival fraction curve of each group was obtained by a nonlinear fitting using Origin 2018, and the SER was expressed using the following formulae:

SER = *D_q_* (control group)/*D_q_* (treated group (2)

*D_q_* = *ln (n)* × *D_0_* (3)

### Comet assay

A total number of 2× 10^5^ MCF-7 cells were seeded in a six-well plate and cultured at incubators for 18 h. After treatment with different forms of drugs for 12 h, the cells in the irradiation group were exposed to 6 Gy IR. After radiation, approximately 3,000 cells were suspended in low-melting-point (LMP) agarose (0.75%) at 37 °C and layered onto frosted slides, which were pre-coated with normal-melting-point agarose (NMP) (Sigma-Aldrich, Lisbon, Portugal) at 4 °C. Subsequently, the slides were incubated in freshly prepared and pre-cooling lysis buffer (2.5 M NaCl, 100 mM Na_2_EDTA, 10 mM tris-HCl, 10% DMSO and 1% Triton X-100) for 2.5 h. Following this, the slides were immersed in a horizontal gel electrophoresis tank for 20 min, which were filled with a pre-cooling electrophoresis buffer (1 mM Na_2_EDTA and 300 mM NaOH), and then were electrophoresed in the same buffer at 30 V voltage for 20 min. After the electrophoresis, the slides were neutralized with tris-HCl (pH =7) for 20 min, and then stained with Gel Signal™Red (2 μg/mL). In addition, the slides needed to be rinsed with PBS before changing the liquid and were kept at 4 °C continually. Finally, these slides were visualized at 40× magnification using a fluorescence microscope (Axioplan, Carl-Zeiss, Germany) and estimated the DNA tail was estimated using the Comet Assay Software Project (CASP).

### Immunofluorescence staining

MCF-7 cells with the number of 2×10^5^ were seeded in a confocal dish and cultured for 18 h. After treatment with different forms of drugs for 12 h, the cells in the irradiation group were exposed to 6 Gy IR. After being kept in an incubator for another 1 h, the cells in each group were fixed in 4% paraformaldehyde solution for 15 min at room temperature. Then the cells were blocked with 5% defatted milk powder in PBS for 1 h, followed by incubated for 15 min in ice methanol at -20 °C. Following this, the cells were incubated with mouse anti-γ-H2AX antibody at 4 °C overnight. After washing with PBS, the cells were subsequently incubated with Rhodamine-conjugated anti-mouse secondary antibody for 1 h in dark. Finally, the fluorescence signal was acquired using a fluorescence microscope after the cells were stained with DAPI for 20 min.

### *In vivo* anti-tumor evaluation

MCF-7 cells (1×10^7^ cells per mouse) were subcutaneously injected into the breast regions of six-week-old nude mice to establish the tumor model. When the tumor volume grew to approximately 100 mm^3^, all the mice were randomly divided into eight groups with seven mice in each group. Except for the control groups, all groups were intravenously administrated with 200 μL of LND-Pep-cyclen nanofibers, free LND, and mixture of free LND and free cyclen (5 mg/kg LND equivalent and 2 mg/kg cyclen equivalent), respectively. After 12 hours, the mice in the chemo-radiotherapy group were exposed to 6 Gy γ-rays. To evaluate the therapeutic efficacy, the tumor size and the body weight were recorded daily. The tumor volumes were measured using a vernier caliper and then calculated using the formula: (width^2^×length)/2. On the 14^th^ day, all the mice were euthanized when the tumor volume in the PBS control group reached approximately 1500 mm^3^. The tumors and normal tissues including heart, liver, spleen, lung and kidney in each group were carefully collected, photographed, and then fixed in 4% form aldehyde for histological analysis.

### Statistical analysis

The SPSS Statistics 19.0 software was used to perform the statistical analysis. Quantitative comparisons of the parameters among two or more groups were made by one-way analysis of variance F-test. Data were expressed as mean ± standard deviation. P-value < 0.05 meant statistically significant.

## Supplementary Material

Supplementary methods and figures.

## References

[1] Martinez-Outschoorn UE, Peiris-Pages M, Pestell RG, Sotgia F, Lisanti MP (2017). Cancer metabolism: a therapeutic perspective. Nat Rev Clin Oncol.

[2] Vander Heiden MG (2011). Targeting cancer metabolism: a therapeutic window opens. Nat Rev Drug Discov.

[3] DeBerardinis RJ, Chandel NS (2020). We need to talk about the Warburg effect. Nat Metab.

[4] Lu J, Tan M, Cai Q (2014). The Warburg effect in tumor progression: mitochondrial oxidative metabolism as an anti-metastasis mechanism. Cancer Lett.

[5] Lu J (2019). The Warburg metabolism fuels tumor metastasis. Cancer Metast Rev.

[6] Vander Heiden MG, Cantley LC, Thompson CB (2009). Understanding the Warburg effect: the metabolic requirements of cell proliferation. Science.

[7] Kolb D, Kolishetti N, Surnar B, Sarkar S, Dhar S (2020). Metabolic modulation of the tumor microenvironment leads to multiple checkpoint inhibition and immune cell infiltration. ACS Nano.

[8] Boroughs LK, DeBerardinis RJ (2015). Metabolic pathways promoting cancer cell survival and growth. Nat Cell Biol.

[9] Jiang W, Luo X, Wei L, Yuan S, Cai J, Jiang X (2021). The sustainability of energy conversion inhibition for tumor ferroptosis therapy and chemotherapy. Small.

[10] Liu X, Li Y, Wang K, Chen Y, Shi M, Zhang X (2021). GSH-responsive nanoprodrug to inhibit glycolysis and alleviate immunosuppression for cancer therapy. Nano Lett.

[11] Li K, Lin C, He Y, Lu L, Cai K (2020). Engineering of cascade-responsive nanoplatform to inhibit lactate efflux for enhanced tumor chemo-immunotherapy. ACS Nano.

[12] Porporato PE, Filigheddu N, Pedro JMB, Kroemer G, Galluzzi L (2018). Mitochondrial metabolism and cancer. Cell Res.

[13] Vasan K, Werner M, Chandel NS (2020). Mitochondrial metabolism as a target for cancer therapy. Cell Metab.

[14] Lu L, Liu G, Lin C, Li K, He T, Zhang J (2021). Mitochondrial metabolism targeted nanoplatform for efficient triple-negative breast cancer combination therapy. Adv Healthc Mater.

[15] Lee J, Yesilkanal AE, Wynne JP, Frankenberger C, Liu J, Yan J (2019). Effective breast cancer combination therapy targeting BACH1 and mitochondrial metabolism. Nature.

[16] Birsoy K, Wang T, Chen WW, Freinkman E, Abu-Remaileh M, Sabatini DM (2015). An essential role of the mitochondrial electron transport chain in cell proliferation is to enable aspartate synthesis. Cell.

[17] Sullivan LB, Gui DY, Hosios AM, Bush LN, Freinkman E, Vander Heiden MG (2015). Supporting aspartate biosynthesis is an essential function of respiration in proliferating cells. Cell.

[18] Momcilovic M, Jones A, Bailey ST, Waldmann CM, Li R, Lee JT (2019). *In vivo* imaging of mitochondrial membrane potential in non-small-cell lung cancer. Nature.

[19] Li M, Song Y, Song N, Wu G, Hao Z, Long J (2021). Supramolecular antagonists promote mitochondrial dysfunction. Nano Lett.

[20] Jeena MT, Palanikumar L, Go EM, Kim I, Kang MG, Lee S (2017). Mitochondria localization induced self-assembly of peptide amphiphiles for cellular dysfunction. Nat Commun.

[21] Tian J, Huang B, Cui Z, Wang P, Chen S, Yang G (2021). Mitochondria-targeting and ROS-sensitive smart nanoscale supramolecular organic framework for combinational amplified photodynamic therapy and chemotherapy. Acta Biomater.

[22] Wang K, Zhu C, He Y, Zhang Z, Zhou W (2019). Restraining cancer cells by dual metabolic inhibition with a mitochondrion-targeted platinum (II) complex. Angew Chem Int Ed Engl.

[23] Deng Y, Song P, Chen X, Huang Y, Hong L, Jin Q (2020). 3-Bromopyruvate-conjugated nanoplatform-induced pro-death autophagy for enhanced photodynamic therapy against hypoxic tumor. ACS Nano.

[24] Shin J, Xu Y, Koo S, Lim JH, Kim JS (2021). Mitochondria-targeted nanotheranostic: harnessing single-laser-activated dual phototherapeutic processing for hypoxic tumor treatment. Matter.

[25] He H, Lin X, Guo J, Wang J, Xu B (2020). Perimitochondrial enzymatic self-assembly for selective targeting the mitochondria of cancer cells. ACS Nano.

[26] Cheng DB, Zhang XH, Gao YJ, Ji L, Hou D, Wang Z (2019). Endogenous ROS-triggered morphology transformation for enhanced cooperative interaction with mitochondria. J Am Chem Soc.

[27] Ni K, Lan G, Veroneau SS, Duan X, Song Y, Lin W (2018). Nanoscale metal-organic frameworks for mitochondria-targeted radiotherapy-radiodynamic therapy. Nat Commun.

[28] Song G, Cheng L, Chao Y, Yang K, Liu Z (2017). Emerging nanotechnology and advanced materials for cancer radiation therapy. Adv Mater.

[29] Tian J, Zhang W (2019). Synthesis, self-assembly and applications of functional polymers based on porphyrins. Prog Polym Sci.

[30] Yu C, Xu H, Ji S, Kwok R, Lam J, Li X (2017). Mitochondrion-anchoring photosensitizer with aggregation-induced emission characteristics synergistically boosts the radiosensitivity of cancer cells to ionizing radiation. Adv Mater.

[31] Li J, Wei Y, Yang X, Wu W, Zhang M, Li M (2020). Rational construction of a mitochondrial targeting, fluorescent self-reporting drug-delivery platform for combined enhancement of endogenous ROS responsiveness. ACS Appl Mater Inter.

[32] He H, Wang J, Wang H, Zhou N, Yang D, Green DR (2018). Enzymatic cleavage of branched peptides for targeting mitochondria. J Am Chem Soc.

[33] Zielonka J, Joseph J, Sikora A, Hardy M, Ouari O, Vasquez-Vivar J (2017). Mitochondria-targeted triphenylphosphonium-based compounds: syntheses, mechanisms of action, and therapeutic and diagnostic applications. Chem Rev.

[34] Modica-Napolitano J, Aprille J (2001). Delocalized lipophilic cations selectively target the mitochondria of carcinoma cells. Adv Drug Deliv Rev.

[35] Yu H, Jin F, Liu D, Shu G, Wang X, Qi J (2020). ROS-responsive nano-drug delivery system combining mitochondria-targeting ceria nanoparticles with atorvastatin for acute kidney injury. Theranostics.

[36] Jiang L, Zhou S, Zhang X, Li C, Ji S, Mao H (2021). Mitochondrion-specific dendritic lipopeptide liposomes for targeted sub-cellular delivery. Nat Commun.

[37] Jean S, Ahmed M, Lei E, Wisnovsky S, Kelley S (2016). Peptide-mediated delivery of chemical probes and therapeutics to mitochondria. Acc Chem Res.

[38] Bazzicalupi C, Bencini A, Lippolis V (2010). Tailoring cyclic polyamines for inorganic/organic phosphate binding. Chem Soc Rev.

[39] Ju Y, Wu J, Yuan X, Zhao L, Zhang G, Li C (2018). Design and evaluation of potent EGFR inhibitors through the incorporation of macrocyclic polyamine moieties into the 4-anilinoquinazoline scaffold. J Med Chem.

[40] Ren C, Wang Z, Zhang X, Gao J, Liu J (2021). Construction of all-in-one peptide nanomedicine with photoacoustic imaging guided mild hyperthermia for enhanced cancer chemotherapy. Chem Eng J.

[41] Sun C, Wang Z, Yang K, Yue L, Cheng Q, Ma Y (2021). Polyamine-responsive morphological transformation of a supramolecular peptide for specific drug accumulation and retention in cancer cells. Small.

[42] Wang Q, Xiao M, Wang D, Hou X, Gao J, Liu J (2021). *In situ* supramolecular self-assembly of Pt(IV) prodrug to conquer cisplatin resistance. Adv Funct Mater.

[43] Xu H, Li X, Ding D (2018). Supramolecular nanofibers of curcumin for highly amplified radiosensitization of colorectal cancers to ionizing radiation. Adv Funct Mater.

[44] Sun B, Chang R, Cao S, Yuan C, Zhao L, Yang H (2020). Acid-activatable transmorphic peptide-based nanomaterials for photodynamic therapy. Angew Chem Int Ed Engl.

[45] Wang Z, Shang Y, Tan Z, Li X, Li G, Ren C (2020). A supramolecular protein chaperone for vaccine delivery. Theranostics.

[46] Cheng G, Zhang Q, Pan J, Lee Y, Ouari O, Hardy M (2019). Targeting lonidamine to mitochondria mitigates lung tumorigenesis and brain metastasis. Nat Commun.

[47] Nath K, Guo L, Nancolas B, Nelson D, Shestov A, Lee S (2016). Mechanism of antineoplastic activity of lonidamine. Biochim Biophys Acta.

[48] Wu C, Wang C, Zheng Y, Zheng Y, Liu Z, Xu K, Triple enzyme-regulated molecular hydrogels for carrier-free delivery of lonidamine Adv Funct Mater. 2021;2104418.

[49] Sun C, Wang Z, Yue L, Huang Q, Cheng Q, Wang R (2020). Supramolecular induction of mitochondrial aggregation and fusion. J Am Chem Soc.

[50] Liu Y, Zhang X, Zhou M, Nan X, Chen X, Zhang X (2017). Mitochondrial-targeting lonidamine-doxorubicin nanoparticles for synergistic chemotherapy to conquer drug resistance. ACS Appl Mater Inter.

[51] Cui L, Gouw A, LaGory E, Guo S, Attarwala N, Tang Y (2021). Mitochondrial copper depletion suppresses triple-negative breast cancer in mice. Nat Biotechnol.

[52] Perelman A, Wachtel C, Cohen M, Haupt S, Shapiro H, Tzur A (2012). JC-1: alternative excitation wavelengths facilitate mitochondrial membrane potential cytometry. Cell Death Dis.

[53] Codony V, Tavassoli M (2021). Hypoxia-induced therapy resistance: Available hypoxia-targeting strategies and current advances in head and neck cancer. Transl Oncol.

[54] Wu H, Lin J, Liu P, Huang Z, Zhao P, Jin H (2016). Reactive oxygen species acts as executor in radiation enhancement and autophagy inducing by AgNPs. Biomaterials.

