# Supplementary material for: Mitochondrion-targeted supramolecular “nano-boat” simultaneously inhibiting dual energy metabolism for tumor selective and synergistic chemo-radiotherapy
Source: Theranostics. 2022 Jan 1;12(3):1286–302. doi: 10.7150/thno.67543 (PMC8771563; doi:10.7150/thno.67543)
Supplement: Supplementary file 1 — Supplementary methods and figures. [file thnov12p1286s1.pdf]

Supporting Information

Mitochondrion-targeted supramolecular "nano-boat" simultaneously inhibiting dual energy metabolism for tumor selective and synergistic chemo-radiotherapy

Jie Gao[1#], Zhilong Wang[1#], Qingxiang Guo[1#], Huan Tang[2], Zhongyan Wang[1], Cuihong Yang[1], Huirong Fan[1], Wenxue Zhang[2✉], Chunhua Ren[1✉] , Jianfeng Liu[1✉]

1. Key Laboratory of Radiopharmacokinetics for Innovative Drugs, Institute of Radiation Medicine, Chinese Academy of Medical Sciences & Peking Union Medical College, Tianjin 300192, P. R. China

2. Radiation Oncology Department, Tianjin Medical University General Hospital, Tianjin 300052, P. R. China

# These authors contributed equally to this work.

✉Corresponding authors: Jianfeng Liu and Chunhua Ren, Key Laboratory of Radiopharmacokinetics for Innovative Drugs, Institute of Radiation Medicine, Chinese Academy of Medical Sciences & Peking Union Medical College, Tianjin 300192, P. R. China, E-mail: liujianfeng@irm-cams.ac.cn, renchunhua@irm-cams.ac.cn; Wenxue Zhang, Radiation Oncology Department, Tianjin Medical University General Hospital, Tianjin 300052, P. R. China, E-mail: wenxuezhang@tmu.edu.cn.

Synthesis of cyclen derivatives

*(i) Synthesis of compound **1**.* Di-tert-butyl dicarbonate (24 mL, 104.5 mmol) in 10 mL DCM was added in dropwise to the stirred solution of cyclen (6 g, 34.8 mmol), and DIEA (24 mL, 104.5 mmol) were dissolved in 40 mL DCM, stirred 5 h at room temperature. The reaction mixture was concentrated under vacuum. The crude product was purified by silica gel column chromatography to afford compound ***1*** in 86% yield as a colorless oil. ^{1}H NMR (600 MHz, DMSO-d_6) δ 3.60 – 3.49 (m, 4H), 3.29 – 3.13 (m, 8H), 2.69 – 2.58 (m, 4H), 1.43 – 1.31 (m, 27H).

*(ii) Synthesis of compound **2**.* Compound ***1*** (14.1 g, 29.8 mmol), methyl bromoacetate (2.9 mL, 29.8 mmol) and K_2CO_3 (8.3 g, 59.7 mmol) were suspended in 50 mL DMF, stirred at 70 °C overnight. The reaction mixture was concentrated under vacuum. The crude product was purified by silica gel column chromatography to afford compound ***2*** in 87% yield as a colorless oil. ^{1}H NMR (600 MHz, DMSO-d_6) δ 3.62 (s, 3H), 3.59 (s, 2H), 3.47 – 3.18 (m, 12H), 2.85 – 2.76 (m, 4H), 1.42 – 1.32 (m, 27H).

*(iii) Synthesis of compound **3**.* Compound ***2*** (14.2 g, 26.1 mmol) was dissolved in 30 mL THF, then 30 mL 2N aqueous sodium hydroxide was added. The resulting mixture was stirred at 60 °C overnight, and then was adjusted pH to 6 using 1 N hydrochloric acid after THF was removed under reduced pressure. The resulting reaction mixture was extracted 3 times with ethyl acetate to obtain the organic layer, which was washed with saturated NaCl solution, dried with anhydrous Na_2SO_4, concentrated under vacuum. The crude product was dissolved in 50 mL DMF, then HBTU (7.7 g, 20.2 mmol), N-Hydroxysuccinimide (2.3 g, 20.2 mmol) were added, stirred overnight

at room temperature. The solvent was removed under vacuum. The crude product was dissolved in ethyl acetate, washed with saturated NaCl solution, dried with anhydrous Na_2SO_4, concentrated under vacuum and was used to conjugate on peptide without further purification.

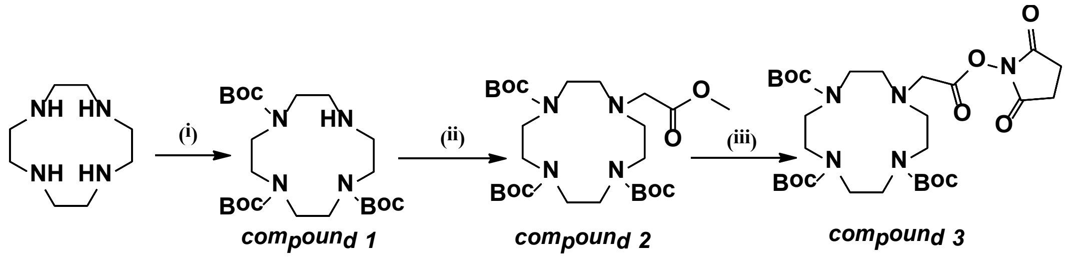

Figure S1 Synthesis routes of the active ester of cyclen derivatives.

Synthesis of LND-GFFYK-cyclen (LND-Pep-cyclen)

The synthesis routes of LND-Pep-cyclen was shown in Figure S2. Firstly, LND-Pep was prepared by standard solid phase peptide synthesis (SPPS) using the corresponding N-Fmoc protected amino acids with side chains properly protected and 2-chlorotrityl chloride resin. LND was regarded as an amino acid as well, and was directly added into the solid phase reaction. During the condensation reaction, HBTU and DIEA were used as coupling agent and catalyst, respectively. The final product of LND-GFFYK (LND-Pep) was cleaved from the resin using a mixture of TFA (19 mL), TIS (0.5 mL), and H_2O (0.5 mL) for 30 min. The reaction mixture was concentrated under reduced pressure, followed by adding diethylether to obtain the precipitate. After drying, the crude product was used for next step without further purification. LND-Pep (200 mg, crude product), compound *3* (300 mg, crude product), DIEA (0.8 mL), was dissolved in DMF, stirred overnight at room temperature. The solvent evaporated to dryness, then re-dissolved in TFA/DCM (95/5) and stirred 3 hours

followed by concentrated under vacuum. The crude product was purified by HPLC and then frozen dried to obtain pure product of LND-Pep-cyclen. NBD-Pep-cyclen was synthesized using the same method.

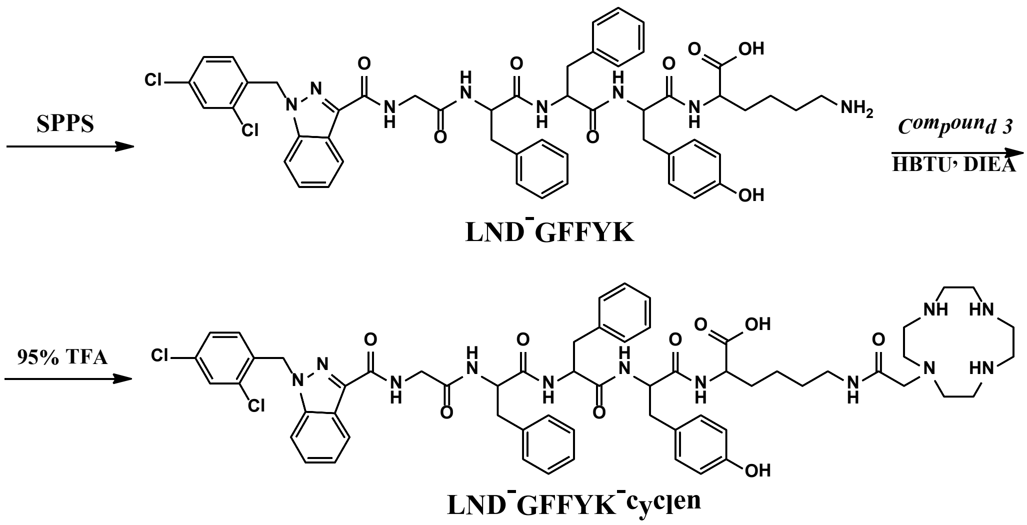

Figure S2 Synthesis routes of the peptide derivatives of *LND-GFFYK-cyclen*.

Synthesis of NBD-GFFYK-cyclen (NBD-Pep-cyclen)

Firstly, the compound of NBD derivative was synthesized according to the following method: under the protection of nitrogen, β-alanine (98 mg, 1.1 mmol) and Na_2CO_3 were added to the mixed solution of methanol and water, and 5 mL 4-chloro-7-nitrobenzo-2-oxa-1 (200 mg, 1 mmol) was slowly injected into the solution. After stirred at room temperature for 5 hours, methanol was removed by vacuum rotary evaporation, and the pH value of the remaining solution was adjusted to 1~ 2 with hydrochloric acid. Next, the crude product was extracted twice with anhydrous ether, and then extracted with dichloromethane twice. After the organic solvent phase was collected and concentrated in vacuum, the crude product could be directly used for the solid phase reaction to obtain the peptide of NBD-GFFYK (NBD-Pep). The final product of NBD-GFFYK-cyclen (NBD-Pep-cyclen) was

yielded using the same synthesis method of LND-Pep-cyclen.

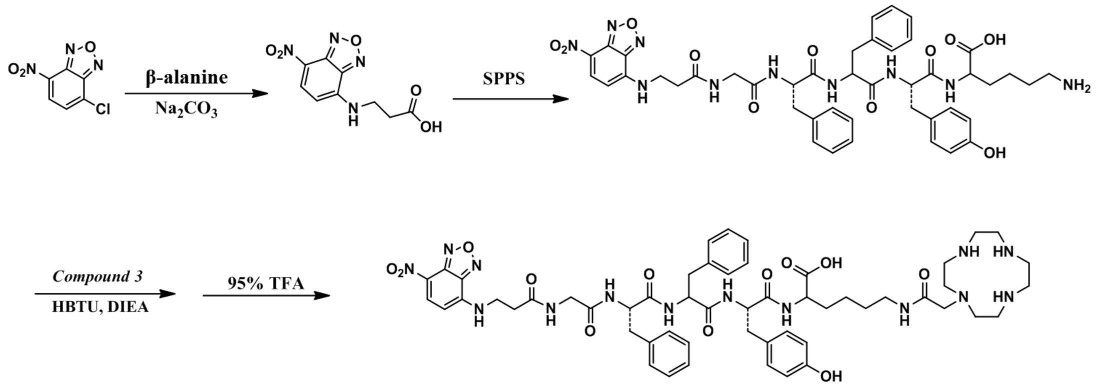

Figure S3 Synthesis routes of the peptide derivatives of *NBD-GFFYK-cyclen*.

Preparation of TEM samples

5 μL sample solution (1 mg/mL) was dropped onto a carbon-coated copper grid for 5 min, and the redundant liquid was gently absorbed from the edge of the grid using a filter paper. Then the sample was stained with 2% uranyl acetate solution for another 5 min in the dark. After removing the redundant dye solution, the carbon-coated copper grids were dried at room temperature for further TEM observation.

Zeta potential

The zeta potential of LND-Pep, NBD-Pep, LND-Pep-cyclen and NBD-Pep-cyclen were measured by dynamic light scattering (BI-200SM). Compounds of 200 μM was diluted with ddH_2O to 1 mL before measurement. Each sample was detected in triplicate.

Circular dichroism (CD) spectra

The secondary structure of LND-Pep-cyclen hydrogel and NBD-Pep-cyclen hydrogel were monitored by a circular dichroism (CD) spectrophotometer (BioLogic, MOS-450, France) system. The self-assembled hydrogel was added into a 0.1 cm quartz spectrophotometer cell (20-C/Q/0.1), then the CD signals were recorded in the 180-280 nm scanning wavelength range. The final spectrogram was obtained after subtracting the PBS solvent background.

Cell culture

Mammary carcinoma cancer cells (MCF-7 and 4T1), and mouse fibroblast cells normal cells (L929) were all maintained in our lab. These cells were cultured in DMEM (MCF-7) and RPMI 1640 medium (4T1 and L929) supplemented with 10% FBS and 1% penicillin-streptomycin in a humidified incubator maintained at 37 °C and 5% CO_2, respectively.

Cytotoxicity and apoptosis analysis

All kinds of cells were seeded in 96-well plates at a density of 5000 cells per well. After 24 h incubation, the cells were treated with a series of pre-determined doses of LND-Pep-cyclen nanofibers, free LND, free cyclen, mixture of free LND and cyclen molecules for 48 h. After adding 10 μL CCK-8 solution to each well, the cells were incubated at 37 °C for another 4 h. OD_{450} was measured using a Thermo Scientific™ Varioskan™ LUX (Thermo, CA, USA), and then the cell viability values

of different groups at different doses were calculated. For apoptosis assay, MCF-7 and L929 cells were cultured in 6-well plates at a density of 2×10^5 cells per well, then were treated with different forms of drug for 48 h. Afterwards, cells were harvested at 1000 rpm for 5 min and then washed with ice-cold PBS twice. Upon being suspended in 100 μL 1× binding buffer, the samples were incubated with 5 μL of FITC-Annexin-V and 5 μL of PI for 15 min at room temperature in the dark. Finally, the cells were added in 100 μL 1× binding buffer blending gently and analyzed by flow cytometry (Becton Dickinson, NJ, USA) within 1 h.

ROS detection

MCF-7 cancer cells and L929 normal cells with a density of 1×10^5 cells/mL were cultured in confocal chambers and 6-well plates at 37 °C, respectively. After 70% confluence, the cells were treated with fresh medium which contained the same concentrations of LND-Pep-cyclen nanofibers, LND, cyclen, mixture of free LND and cyclen molecules for 12 h. Then, followed by washing twice with PBS and incubated with 1 mL DCFH-DA (10 μM) at 37 °C for 30 min in the dark. The cells in 6-well plates were harvested, washed, re-suspended and then loaded on flow cytometry for quantitate analysis. The ROS production images in confocal chambers were collected on CLSM after washed with PBS for three times. Excitation at 488 nm and signal collection at 525 ± 10 nm for DCFH-DA fluorescence.

Mito-ROS detection

The experimental procedures were similar to those of ROS detection. The

difference was that the MCF-7 cells were firstly exposed to 10 μM different treatments, and stained with 5 μM MitoSOX Red Mitochondrial Superoxide Indicator (Invitrogen, M36008) at 37 °C for 10 min.

Western blotting of apoptotic proteins

MCF-7 cells with the number of 1×10^6 were seeded in 10 cm plates and cultured for 18 h before treated with different forms of drug. After co-incubation with LND-Pep-cyclen nanofibers, free LND, free cyclen, mixture of free LND and cyclen, cells in each group were collected and lysed in RIPA lysis buffer (Solarbio, Beijing, China), supplemented with a protease inhibitor (Roche, Indianapolis, IN, USA), according to the protocol provided by the manufacturer. The samples were subsequently centrifuged for 5 min at 14000 rpm, after which the supernatants were collected as whole-cell lysates. In addition, the cytosolic and mitochondrial fractions were isolated from the treated cells using the cell mitochondrial isolation kit (Abcam, Cambridge, UK) according to the manufacturer's recommended protocol. After extraction, the protein levels were quantified with BCA Protein Assay Kit (Beyotime, Beijing, China). Protein samples (100 μg) were loaded in the wells of 12% sodium dodecyl sulfate-polyacrylamide gel electrophoresis (SDS-PAGE). After the separation by gel electrophoresis, the proteins were transferred to PVDF membranes (Millipore, Billerica, MA, USA). The membrane was probed with primary antibodies after being blocked by 5% non-fat milk overnight at 4 °C. Primary antibodies including anti-caspase-3, anti-cleaved caspase-3, anti-cytochrome c (1:1000, Abcam,

Cambridge, UK), anti-Bcl-2 (1:1000, Abcam, Cambridge, UK), anti-smac and anti-GAPDH antibody. After washing with 1 ×TBST, membranes were incubated with corresponding secondary antibody for 1 h at room temperature. The membranes were washed 3 times by 1 ×TBST and illuminated by an enhanced chemiluminescent (ECL) kit (Pierce™ ECL Western Blotting Substrate, Thermo Scientific, Rockford, IL, USA) on a Bio-Rad film (Sigma-Aldrich, St. Louis, MO, USA).

Bio-compatibility evaluation

Six-week-old BALB/c mice were randomly assigned to two groups and intravenously administrated with 200 μL of LND-Pep-cyclen nanofibers (20 mg/kg) and PBS at 0 day, 7 day, 14 day, 21 day. All the mice were sacrificed at the last time point, followed by collecting the whole body blood for the hematology and blood biochemical detection.

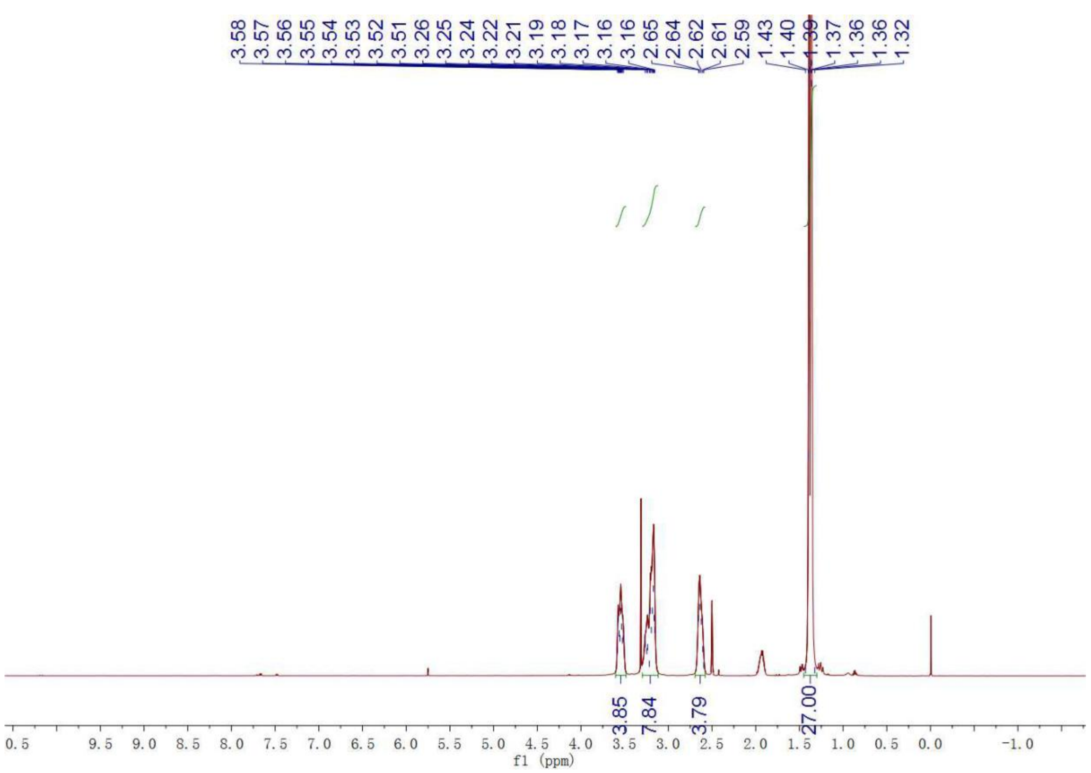

Figure S4 ^{1}H NMR spectrum of ***compound 1***.

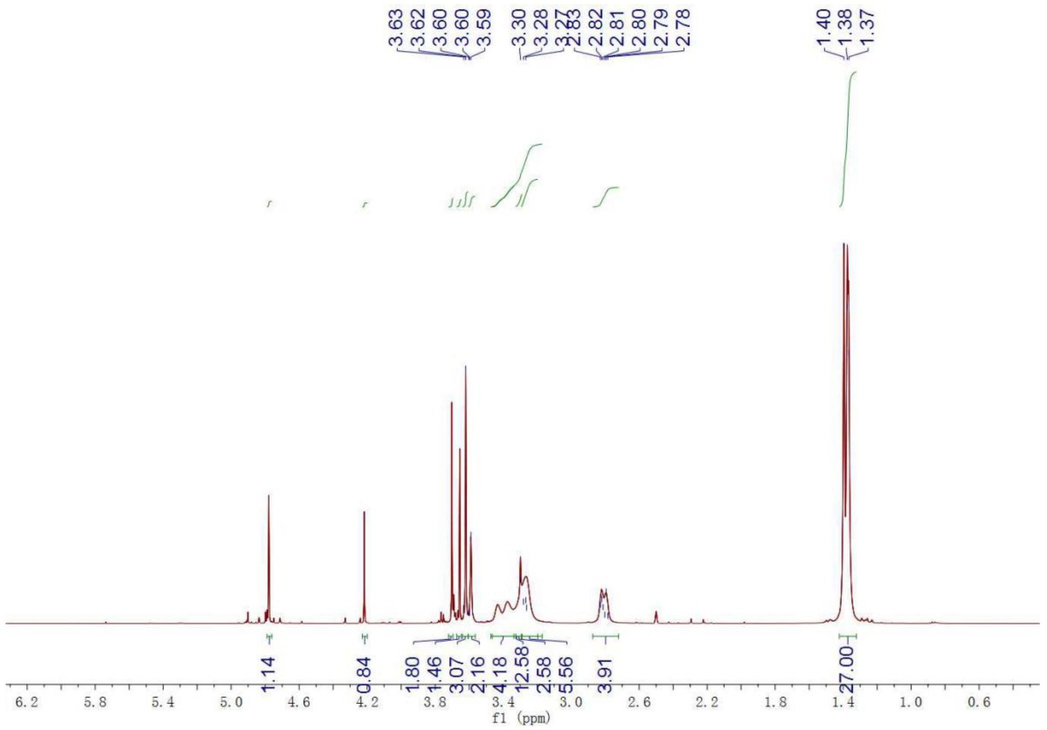

Figure S5 ^{1}H NMR spectrum of ***compound 2***.

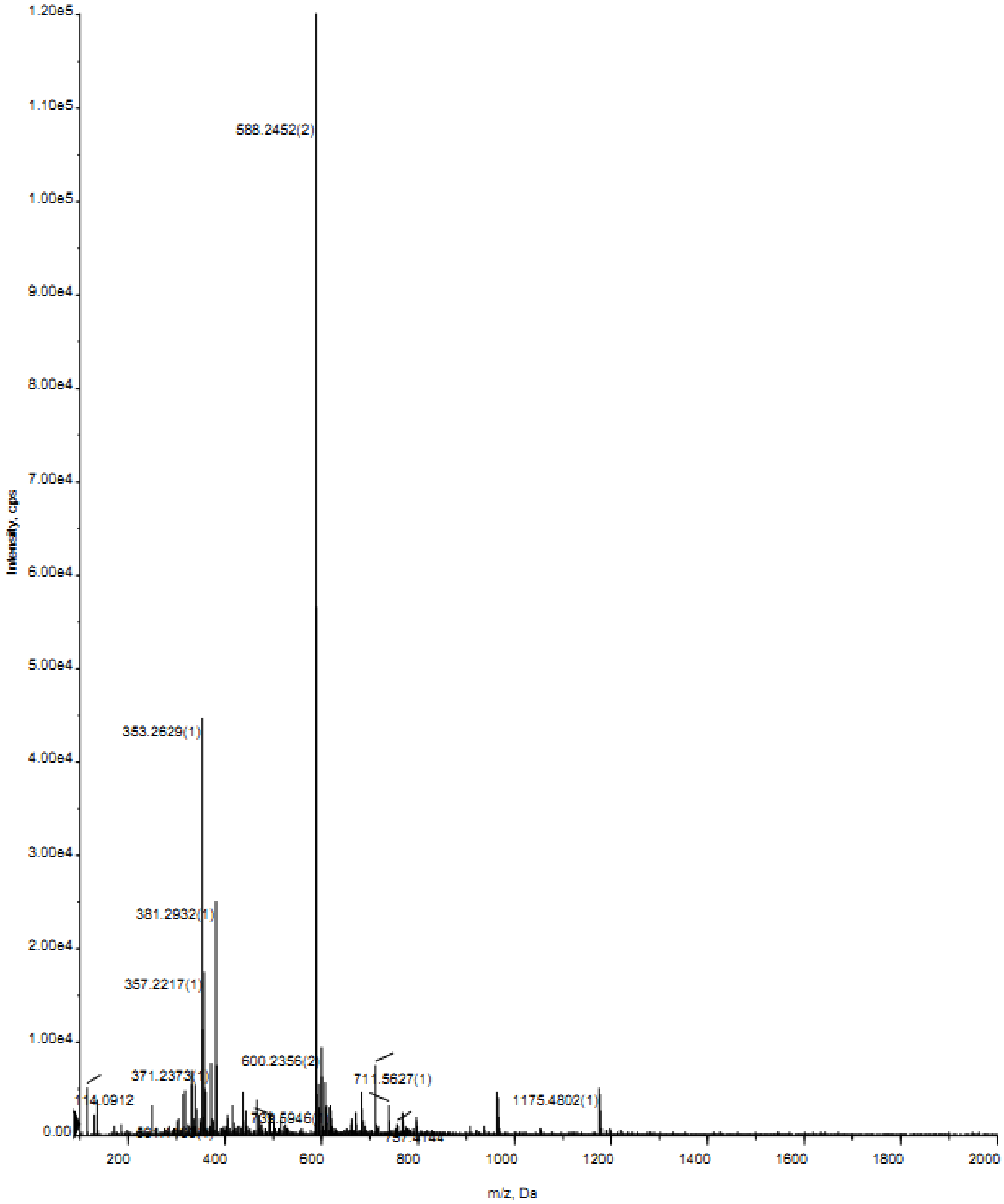

Figure S6 High resolution mass spectrum of *LND-GFFYK-cyclen.* m/z calculated: 1174.49 found:588.2452&1175.4802.

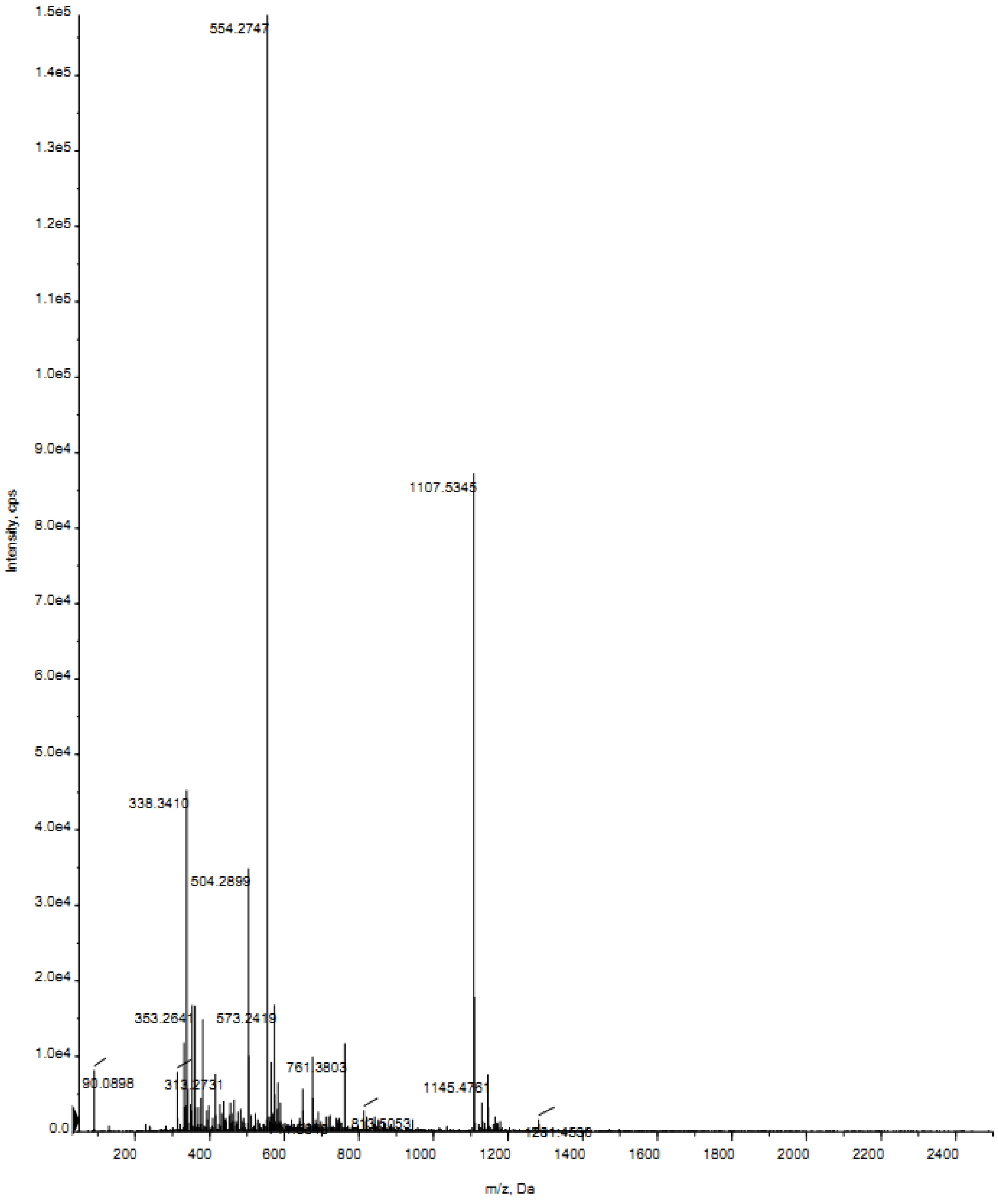

Figure S7 High resolution mass spectrum of *NBD-GFFYK-cyclen.* m/z calculated: 1106.53 found:554.2747&1107.5345.

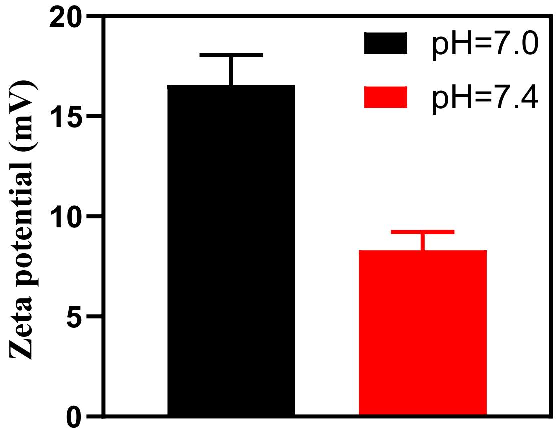

Figure S8 The Zeta potential values of LND-Pep-cyclen at pH 7.0 and 7.4.

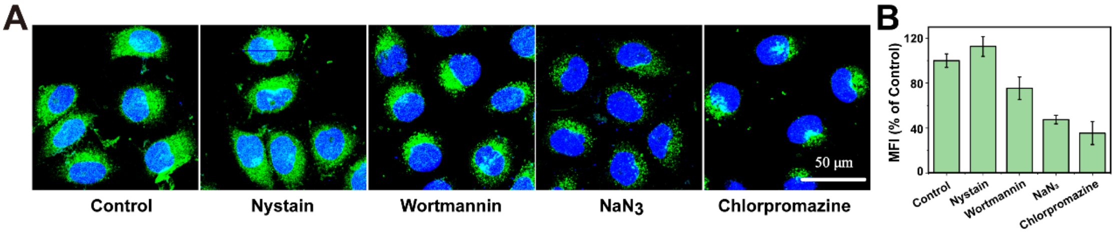

Figure S9 (A) CLSM images of MCF-7 cells treated with NBD-Pep-cyclen nanofibers in the absence and presence of endocytotic inhibitors. (B) Fluorescence quantitative analysis of NBD in each group treated with different endocytotic inhibitors.

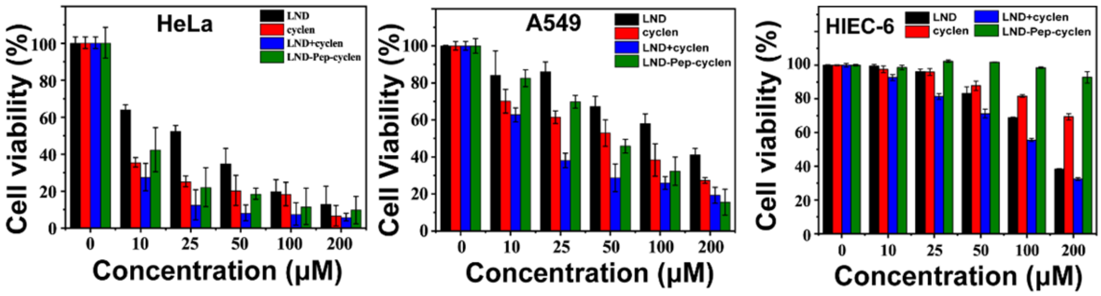

Figure S10 Cell viabilities of HeLa, A549, and HIEC-6 cells after 48 h of incubation with different formulations.

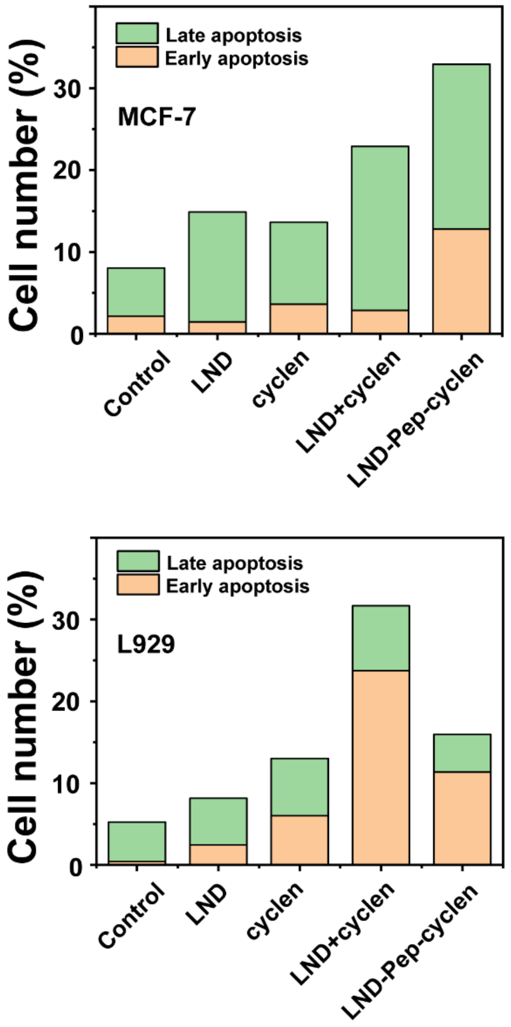

Figure S11 Flow cytometry quantitative analysis of the apoptotic cell number of MCF-7 cells and L929 cells after different treatments.

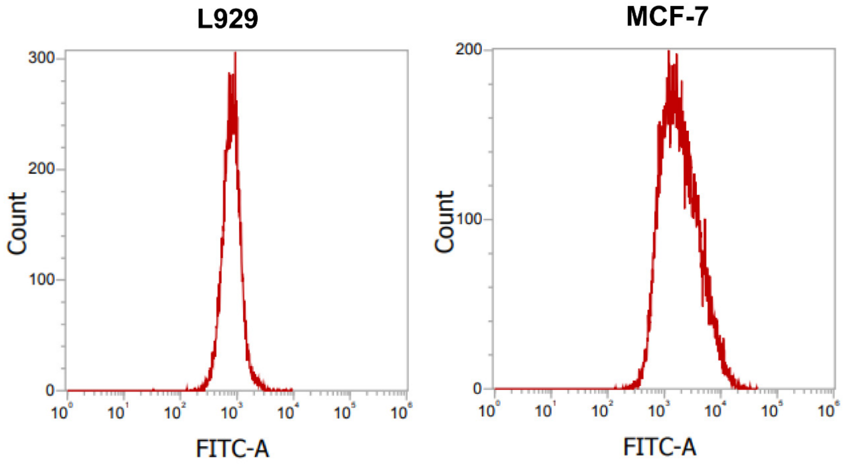

Figure S12 Flow cytometry analysis of mean fluorescent intensity of MCF-7 and L929 cells after treated with NBD-Pep-cyclen nanofibers (50 μM) for 4 h.

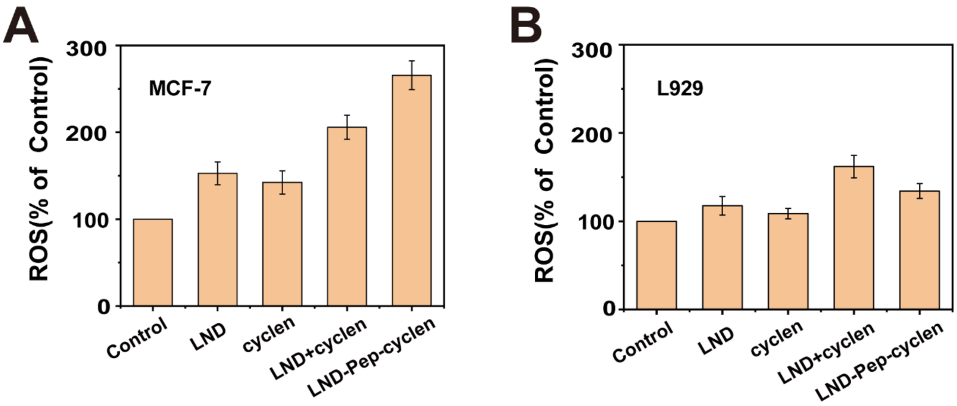

Figure S13 Flow cytometry quantitative analysis of ROS generation of MCF-7 and L929 cells treated with indicated formulations (50 μM)) for 12 h using DCFH-DA assay kit.

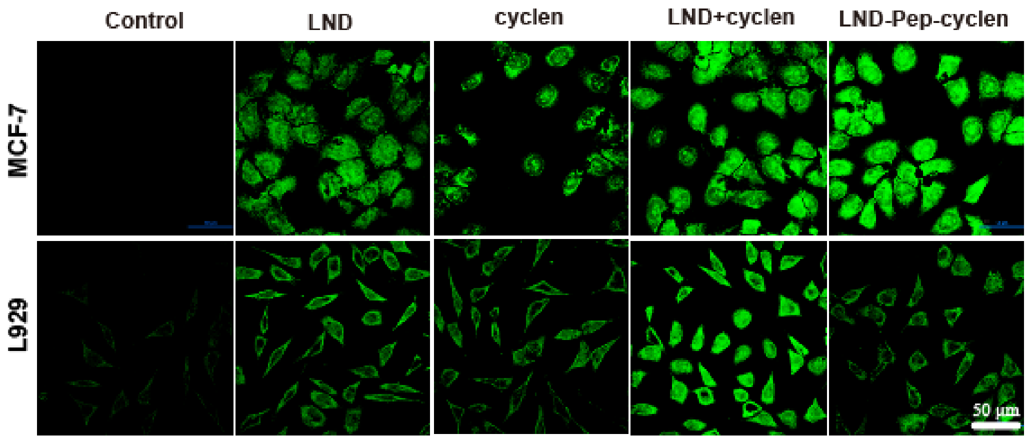

Figure S14 Confocal fluorescence microscopy images of ROS in MCF-7 cells after different treatments at the concentration of 50 μM for 12 h.

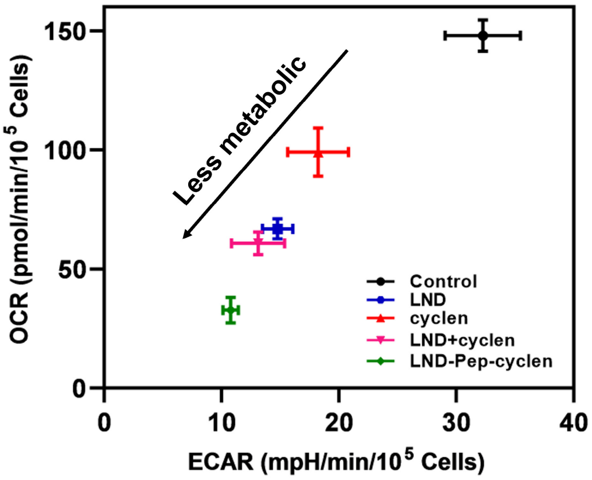

Figure S15 The OCR versus ECAR profile of MCF-7 cells after different treatments.

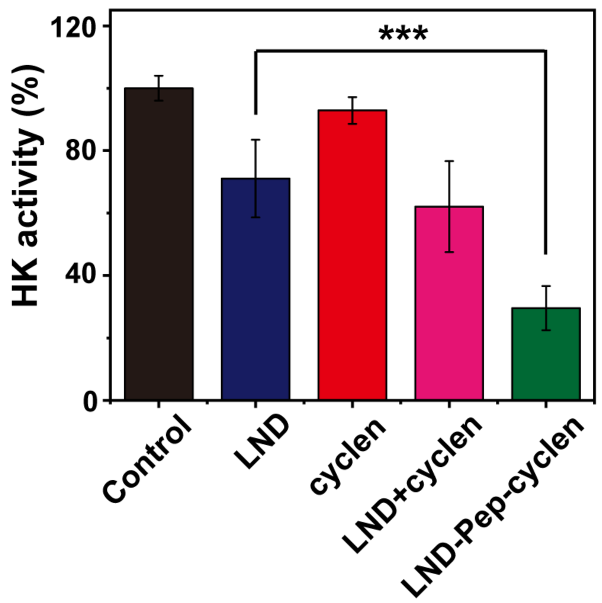

Figure S16 Hexokinase II (HK-II) activity in per cell after MCF-7 cells were treated with different formulations (50 μM) for 24 h. The values of the control group were all set as 100%, and the data was shown as the mean±SD (n = 5). ***P < 0.001.

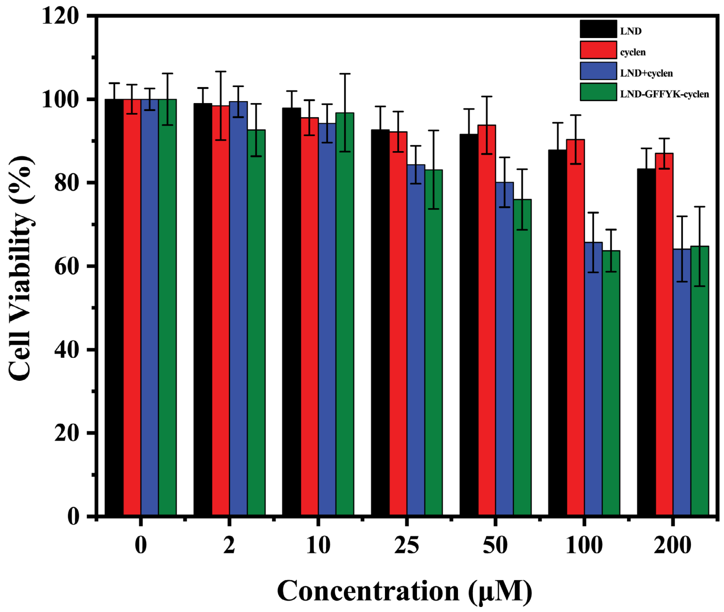

Figure S17 Cell viabilities of MCF-7 cells after 12 h incubation with different treatments at different concentrations.

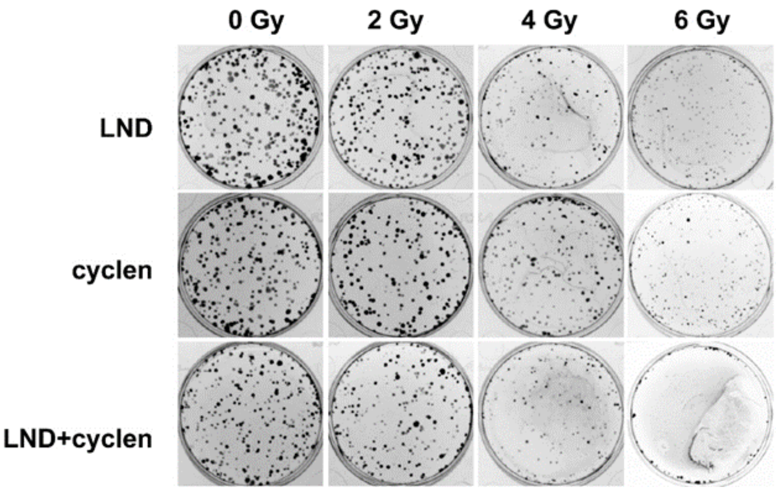

Figure S18 Colony formation photographs of MCF-7 cells treated with the indicated formulations (10 μM) for 12 h, and then exposed to γ-ray with different doses.

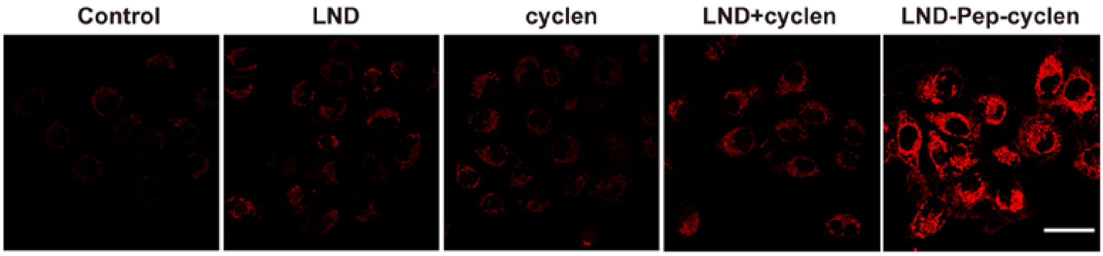

Figure S19 Confocal fluorescence microscopy images of Mito-ROS in MCF-7 cells after different treatments at the concentration of 10 μM for 12 h.Scale bar: 50 μm.

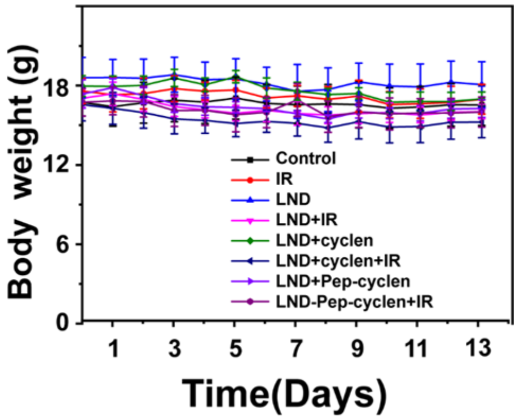

Figure S20 Body weight variation of the mice in different groups.

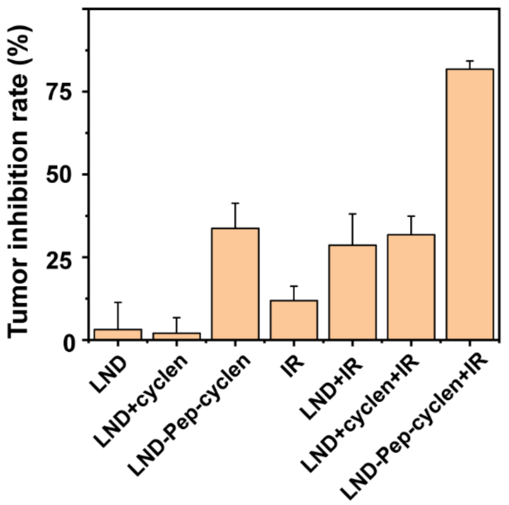

Figure S21 The in vivo tumor inhibition rate of different treatments.

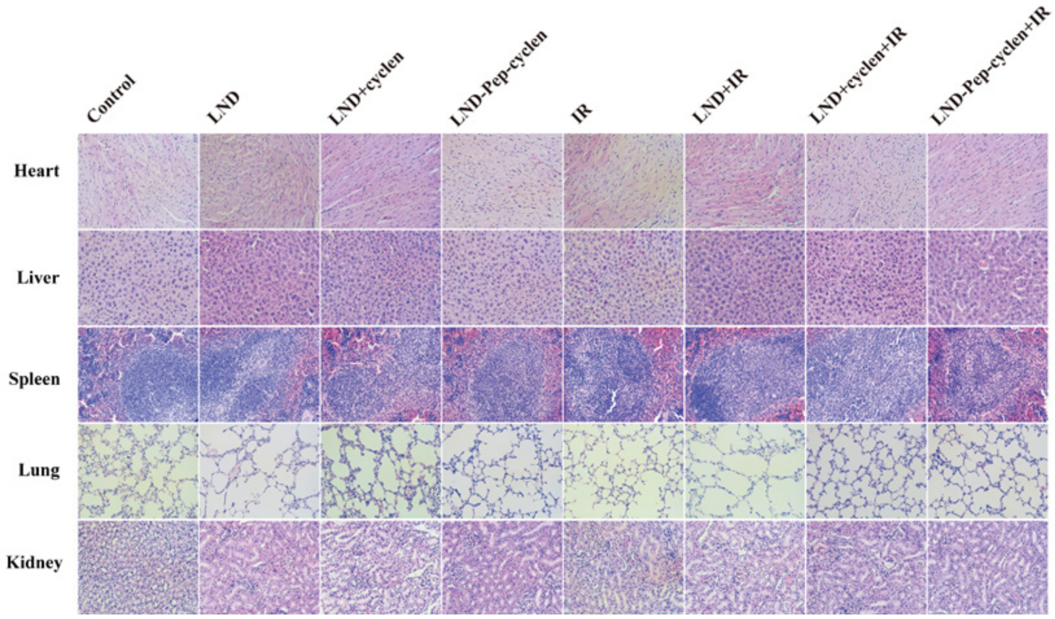

Figure S22 H&E stained tissue sections from the heart, liver, spleen, lung, and kidney of the representative mice in different groups.
